# Solvent Assisted Tuning of Morphology of a Peptide-Perylenediimide Conjugate: Helical Fibers to Nano-Rings and their Differential Semiconductivity

**DOI:** 10.1038/s41598-017-09730-z

**Published:** 2017-08-25

**Authors:** Sahnawaz Ahmed, Bapan Pramanik, K. N. Amba Sankar, Abhinav Srivastava, Nilotpal Singha, Payel Dowari, Arpita Srivastava, Kallol Mohanta, Ananya Debnath, Debapratim Das

**Affiliations:** 1 Department of Chemistry, Indian Institute of Technology Guwahati, Assam, 781039 India; 2 Nanotech Research Innovation and Incubation Centre (NRIIC), PSG Institute of Advanced Studies, Avinashi Road, Coimbatore, 641004 TN India; 3 Department of Chemistry, Indian Institute of Technology Jodhpur, Jodhpur, 342011 India

## Abstract

Understanding the regulatory factors of self-assembly processes is a necessity in order to modulate the nano-structures and their properties. Here, the self-assembly mechanism of a peptide-perylenediimide (**P-1**) conjugate in mixed solvent systems of THF/water is studied and the semiconducting properties are correlated with the morphology. In THF, right handed helical fibers are formed while in 10% THF-water, the morphology changes to nano-rings along with a switch in the helicity to left-handed orientation. Experimental results combined with DFT calculations reveal the critical role of thermodynamic and kinetic factors to control these differential self-assembly processes. In THF, **P-1** forms right handed helical fibers in a kinetically controlled fashion. In case of 10% THF-water, the initial nucleation of the aggregate is controlled kinetically. Due to differential solubility of the molecule in these two solvents, elongation of the nuclei into fibers is restricted after a critical length leading to the formation of nano-rings which is governed by the thermodynamics. The helical fibers show superior semi-conducting property to the nano-rings as confirmed by conducting-AFM and conventional *I-V* characteristics.

## Introduction

Supramolecular organization of monomers *via* noncovalent forces like hydrogen-bonding, π-stacking, electrostatic and hydrophobic interactions have been studied to mimic biological systems and for numerous material applications^1–3^. Control over such supramolecular aggregation requires a thorough mechanistic study which helps to fine tune the assembly process and consequently, the nanostructure of such self-assembled systems^4–7^. Identification of proper molecular structure and functionality is the key to modulate the self-assembly process and generate tailor-made nanostructures^1–7^. In this regard, there is a growing interest in systems capable of assembling into helical arrangements with precise shape and size with nanometer dimensions^8–11^. However, the supramolecular chirality imposed by the chiral center of the monomer on hierarchically organized morphology in solution as well as on surfaces is an extremely multifaceted phenomenon. The growth of such self-assembled systems are influenced by various associated parameters such as solvent, temperature, pH, *etc* ^12–17^. Minor alteration in these parameters often lead to dramatic changes in the supramolecular helicity and consequently to the super structure. Understanding the thermodynamic and kinetic control of these self-organization processes is therefore of utmost importance in order to fabricate and fine tune the nanostructures of these self-organized highly functional systems.

Amino acids and peptides are one of the major natural sources of chirality in nature and has been utilized substantially to construct such supramolecular helical structures in recent past^18–21^. One of the shortest peptide sequence which attracted considerable attention of supramolecular chemists in the recent past is diphenylalanine (PhePhe). This short peptide from the core of Amyloid β peptide self-assembling sequences, was identified through a methodical reductionist approach envisioned to find the minimum recognition motif for self-assembly^22^. Importantly, the so-called “PhePhe” motif with a notably simple chemical structure has recently revealed its full potential in self-assembly. A plethora of scientific reports spanning chemical and nano-technology to medicine and biological applications have recently been published^22–25^. Interestingly, subtle modifications in the chemical structure of the Phe-Phe derivative or minute changes in the experimental conditions are adequate to attain different nanostructures^22–25^. Although the understanding of these systems and the ability to forecast their supramolecular behavior is rapidly developing, yet there is no generalized principle for the design of specific nanostructures.

In this regard, helical nanostructures created by π–systems are of particular interest due to their potential applications in organic electronic devices^26–29^. One such extended π–conjugated ring system is Perylenediimide (PDI). PDIs, with their well characterized self-assembling properties, have recently evolved as important building blocks for functional materials and found their applications in organic-electronic materials such as solar cells, field-effect transistors and light emitting diodes^30–32^. Helical nanostructures formed by PDI-sugar/saccharide conjugates have been reported by Faul *et al*. and found to be influenced by external factors like polarity of the medium^14^. However, except few such examples, not much effort has been made to understand the underlying mechanism behind such differential assembly processes and formation of different nanostructures^12, 14^. We envisioned that the conjugation of the “PhePhe” motif with PDI core will lead to a helical assembly owing to the presence of chiral amino acids as well as a π–stacking unit in the form of PDI. Investigation on the factors influencing the formation and fine tuning of the helical nano-aggregates will certainly be of tremendous importance to gain control over such assemblies. Moreover, correlating the semiconducting properties with the aggregated nano-structures will certainly allow us to create nano-materials with desired material properties.

**Figure 1:**
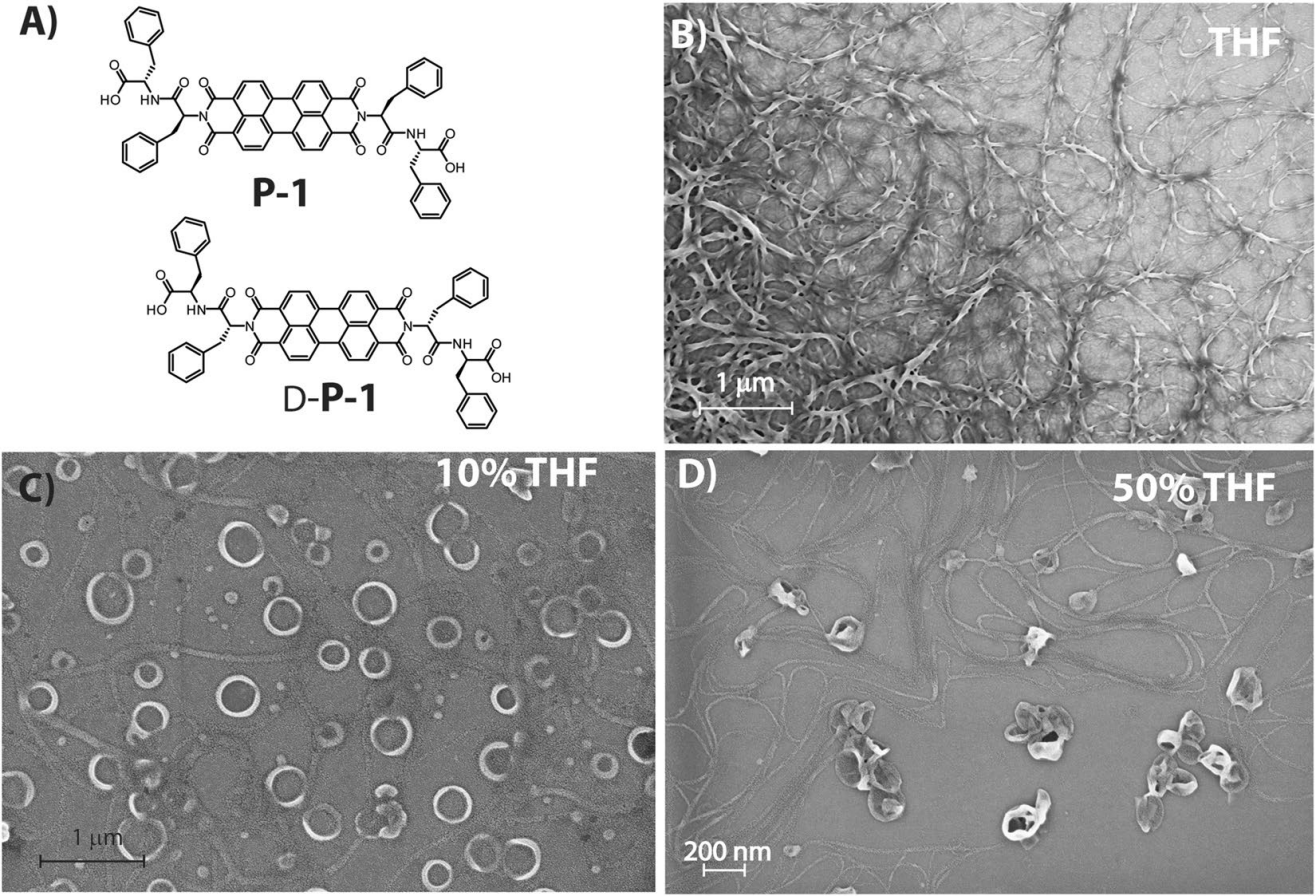
(**A**) Molecular structure of **P-1**; FESEM images of **P-1** (10 μM) nanostructures obtained from THF/water mixed solvents with different volume ratios of (**B**) THF; (**C**) 10% THF and (**D**) 50% THF. All measurements were carried out after 72 h of mixing.

Herein, we report the aggregation behavior of a symmetrical conjugate of “PhePhe” motif and PDI core (**P-1**, Figure 1A). The self-assembly, nanostructure and formation mechanism were studied in tetrahydrofuran (THF) with varying ratio of water. The terminal “PhePhe” dipeptide units provide the supramolecular chirality while the planner symmetrical PDI core is responsible for the well-studied π–π stacking. The combination of these two factors lead to the formation of helical nano-fibers in THF while in presence of higher percentage of water, not only the helicity changed but also the morphology shifted to nano-rings. The detailed study reveals the kinetic and thermodynamic control of the self-assembly processes in different conditions. The presence of PDI moiety leads to the semiconducting behavior of the nanostructures which also depends on the morphology of the assemblies. The insights obtained from this study will help to gain control over the morphology as well as the physical property and applicability of such materials.

## Results and Discussion

The morphologies of **P-1** in THF-water mixed solvents (with different volume ratios at a concentration of 10 μM) were examined by field emission scanning electron microscopy (FESEM). Network of high aspect ratio (3–10 μm) right-handed helical fibers were observed in THF (Figure 1B). Notably, to overrule the possibility of formation of these morphologies due to drying process during the sample preparation, FESEM images of samples at 0.1 and 1 μM concentrations were taken but no clear morphologies could be observed (Figure S13, SI). In a THF-water binary system with high amount of water (90%, above which difficult to solubilize), nano-rings with 150–250 nm diameter were obtained (Figure 1C). Interestingly, in 50% THF, both, fiber and nano-ring like morphologies were observed (Figure 1D). Notably, formation of loop in the fibers to generate the rings were also observed at places. Similar morphologies were also found from transmission electron microscope images (Figure S14, SI).

**Figure 2:**
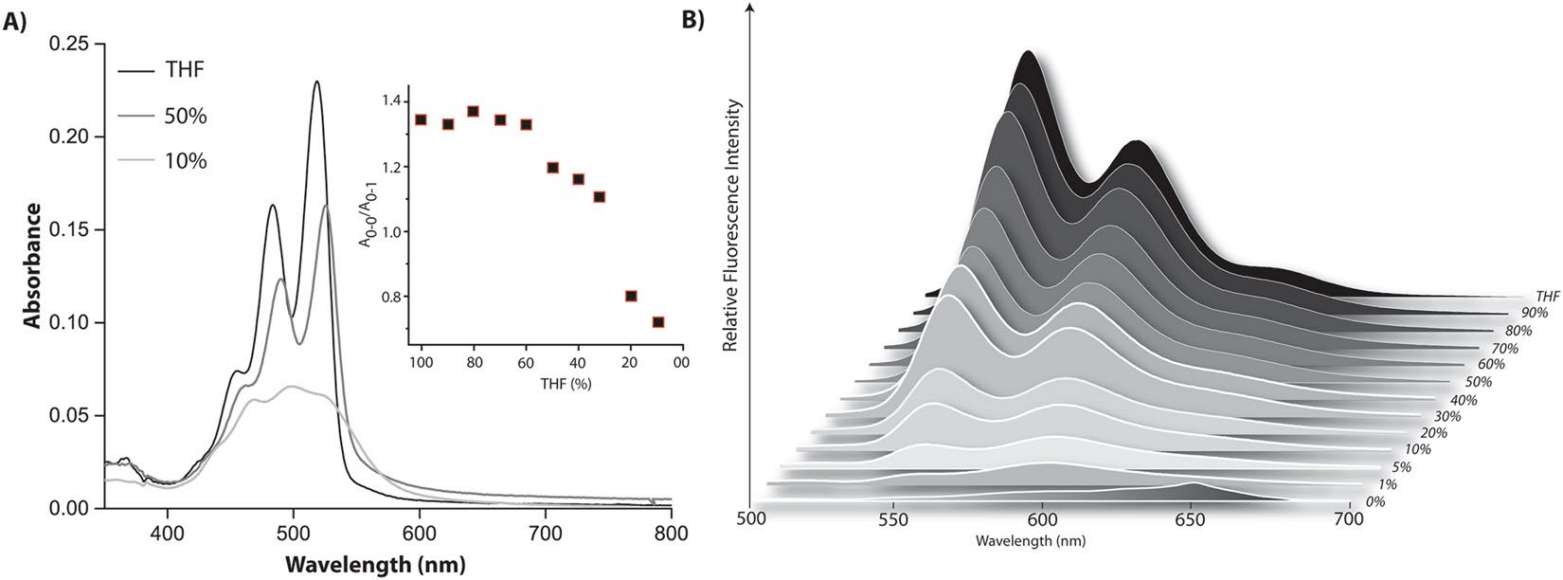
(**A**) Absorption spectra and (**B**) emission spectra of **P-1** (10 µM) in different volume ratio of THF/water under ambient condition. Inset of (**A**) Absorption ratio of the 0–0 and 0–1 transition at different THF content. All measurements were carried out after 72 h of mixing.

To understand the aggregation of **P-1** in these solvent systems, absorption, emission and CD spectra were recorded at various solvent compositions. The absorption spectrum of **P-1** in THF exhibited three characteristic vibronic bands at 517, 482 and 460 nm which are attributed to the 0–0, 0–1 and 0–2 vibrational transitions respectively (Figure 2A and Figure S15, SI)^12, 14, 33–35^. Ratio of the absorption corresponding to the 0–0, 0–1 transitions obtained (A_0–0_/A_0–1_ = 1.44) is far lesser than the same for monomeric species (1.72 at a concentration of 0.1 μM, Figure S16, SI) and higher than the standard value of fully aggregated state (>0.8) suggesting that the part of the population are in aggregated state^36–39^. The aggregation is also supported by the broadening of the signals in the excitation spectra (Figure S17, SI). With gradual increase in water content, a consistent red shift accompanied with decrease in absorbance was recorded for all these transitions. Further, to confirm the aggregation, ^1^H NMR spectra were recorded at different concentration. The signals corresponding to the PDI core as well as Phe units of the conjugate showed a significant up-field shifts (Δ*δ*_PDI_ = 1.39 ppm and Δ*δ*_Phe_ = 0.85 ppm) as we move from 0.1 μM to 10 μM (Figure S18, SI). However, above this concentration, no further change in their respective chemical shifts were observed. The up-field shift signifies the aggregation between the molecules.

Below 20% THF, a drastic change was observed in the absorption spectra as the fine structure is lost and a new shoulder appeared at ~550 nm which is typically a sign of co-facial π–π stacking^12, 36–39^. In 10% THF, A_0–0_/A_0–1_ value dropped to 0.77 which signifies fully aggregated state of molecules in this system^36–39^. The bathochromic shift of the absorption maxima, broadening of peaks along with the appearance of peak at 550 nm indicate the presence of aggregates^12, 14, 40^. The formation of J-aggregates is further supported by emission spectra as shown in Figure 2B. In THF, the emission spectrum is the mirror image of corresponding absorption spectrum with a maxima at 533 nm (Stokes shift of 15 nm). With increase in water content, a bathochromic shift accompanied by quenching of the emission was observed indicating the onset of aggregation. Below 20% THF, the emission is completely lost and a new band appeared at 610 nm. Further information about the aggregation obtained from the appearance of structure-less broad band centered at ~540 nm in the excitation spectra collected at 600 nm (Figure S19, SI).

**Figure 3:**
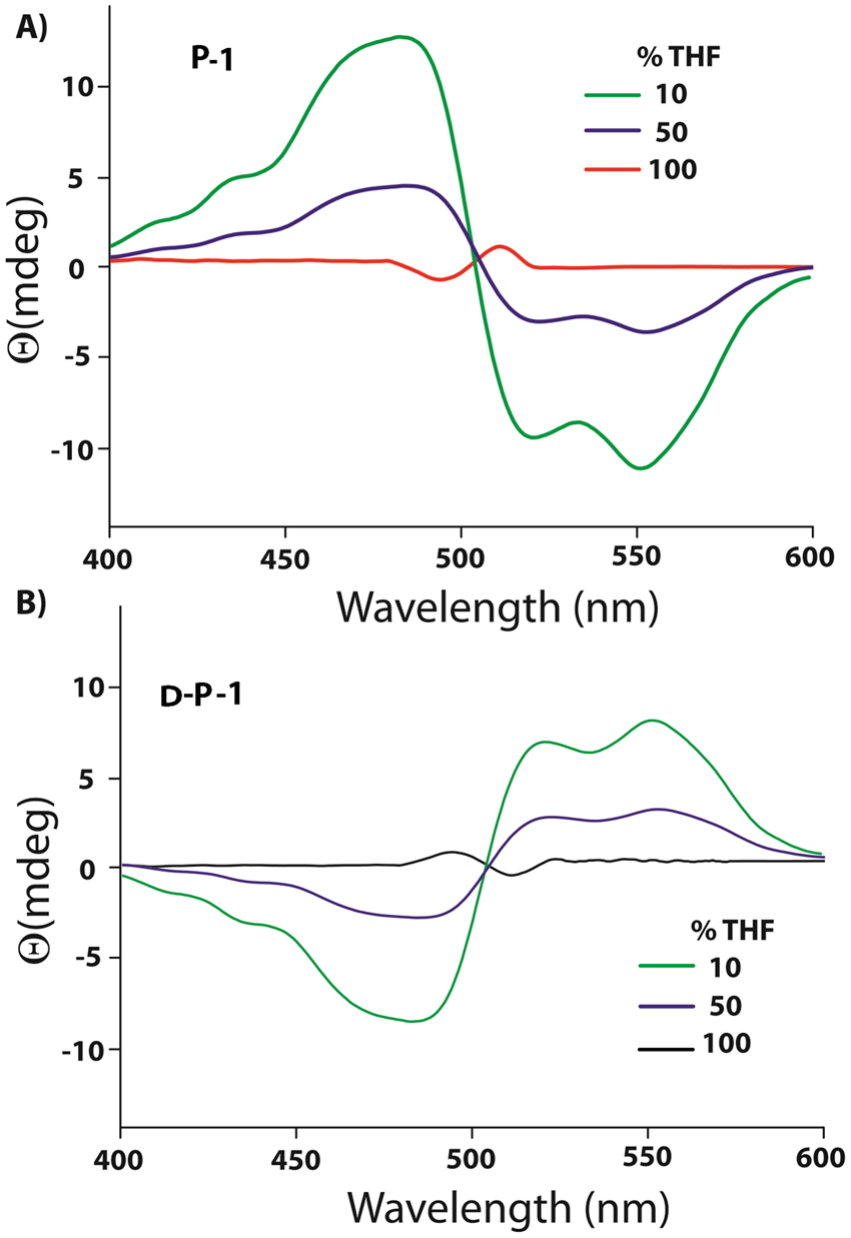
CD spectra of **P-1** (10 µM) in different volume ratio of THF/water under ambient condition. All measurements were carried out after 72 h of mixing.

The circular dichroism (CD) data showed intense cotton effects for **P-1 **in 10% THF. As shown in Figure 3, the CD spectrum showed a bisignate CD signal with a positive signal at 482 nm and negative signals at 522 and 550 nm (crossover at 502 nm). In order to avoid any artifact, the spectra were recorded three times and the signals arising from blank were subtracted. The bisignate CD signal observed in this case indicates aggregated arrangement of **P-1 **where the transition dipoles are oriented in a helical manner^14, 41, 42^. The positive/negative bisignate signal with increasing wavelength suggests that compound **P-1 **adopted a left-handed helical arrangement in this solvent composition^14, 41, 42^. Similar CD signals, though comparatively lower in intensity, were observed in case of 50% THF.

Interestingly, in THF, though the crossover point remains the same, negative to positive bisignate signals were observed along with decrease in intensity. The reversal of the positive and negative signals indicates the change in the helicity from left-handed to right handed orientation. Notably, this is consistent with the right handed helical fibers obtained from THF. The D-analogue (D-**P-1**) of the compound, with D-PhePhe units, showed the mirror image CD signals in each case. The morphologies were also consistent with the CD-signals as left-handed helical fibers were formed in THF (Figures 3 and S20, SI) while in 10% THF, nano-rings were observed.

**Figure 4:**
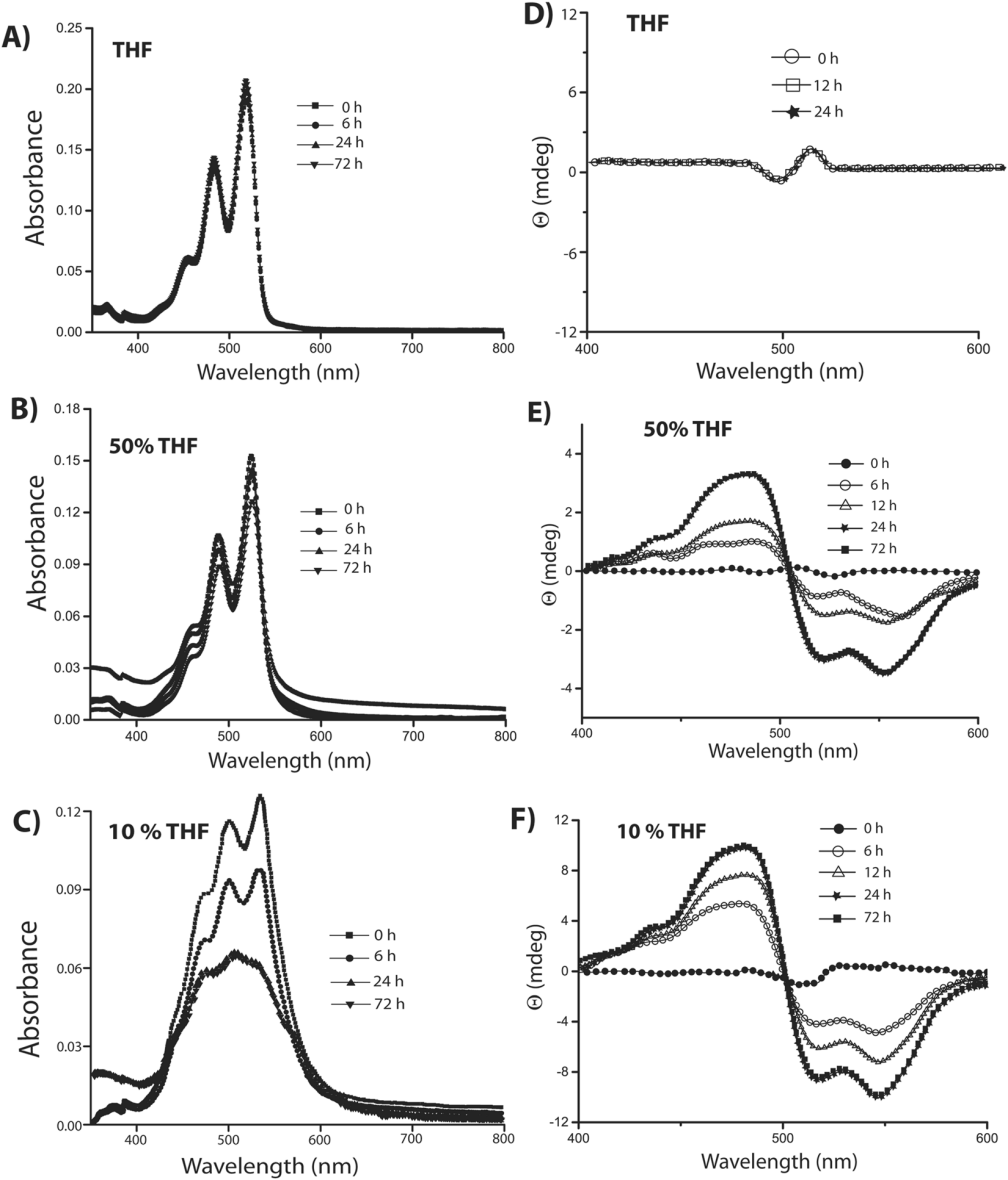
Time dependent (**A**–**C**) absorption and (**D**–**F**) CD spectra of **P**-**1** (10 μM) in THF, 50% THF and 10% THF mixed solvent systems respectively.

To investigate the self-assembly mechanism in these solvent systems, time-dependent absorption and CD spectra of **P-1**in different THF-water compositions were recorded (Figure 4). Interestingly, in case of 10% THF, initially a low intensity bisignate signal was observed. The intensity was drastically enhanced over a period of 24 h and a clear bisignate CD signal with a positive peak at 482 nm and negative signals at 522 and 550 nm were observed (with a crossover at 502 nm). To ensure that the enhancement in the CD is a time dependent process even at the initial stage, the CD spectra were recorded at a 30 minutes interval for first 6 h which show a continuous enhancement of the signal during this period as well (Figure S21, SI). Similarly, in the absorption spectra, the absorption ratio of the 0–0 and 0–1 transitions was observed to be ~1.30 initially. The ratio dropped to 1.1 within a period of 6 h. The absorption ratio dropped further to 0.77 after 24 h and remained unchanged after that. The decrease in the absorption ratio was accompanied with a broadening of the spectra. The saturation value of 0.77 is similar to the aged sample as shown in Figure 2. The observed results suggest that, even at the nucleation stage, **P-1** adopted a left-handed helical arrangement and the nucleation was a kinetically controlled process. With time, the aggregation proceeds in a thermodynamically controlled fashion^12, 14^.

**Figure 5:**
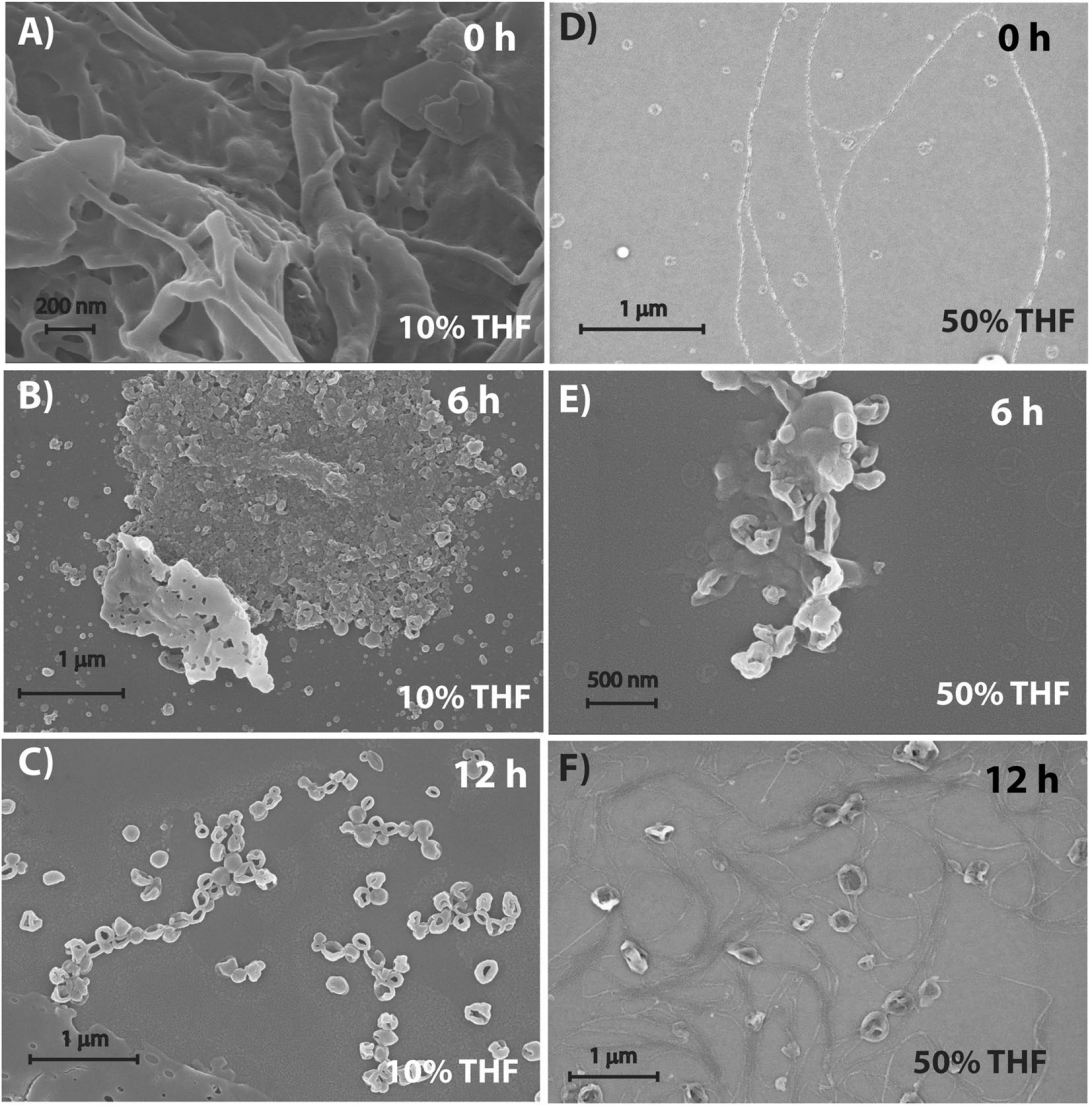
FESEM images of **P-1** (10 μM) nanostructures obtained from 10% THF and 50% THF mixed solvents at different time intervals.

The thermodynamic control was further elucidated by recording the FESEM images at different time intervals (Figure 5A–C). At initial stage (0 h), though no clear morphology could be found, after 6 h, some aggregated structures could be observed. In case of the 12 h aged sample, the formation of ring like structures started appearing. After 24 h, the morphology was very similar to that shown in Fig. 1B and uniform nano-rings were found all over the surface.

However, in THF, neither did the absorption nor did the A_0–0_/A_0–1_ ratio (remained constant at 1.44) change with time up to 72 h (Figure 4). Similarly, no observable change was monitored in the CD signal over a period of 72 h. The morphology study showed right-handed helical fibers from the very initial stage and did not change with time. These observations signify that the aggregation in THF, takes place in a kinetically controlled process^12, 14^.

The case of 50% THF system was also similar to that of the 10% THF. The absorption spectra showed a decrease in absorption with time. The absorption ratio for the 0–0 and 0–1 transitions dropped from 1.33 to a saturation value of 1.21 within 24 h suggesting maximum aggregation after this time. The system showed a weak CD-signal at the initial state and interestingly, showed multiple bisignate signals with crossover points at 482 nm and 521 nm. However, after 6 h, the pattern changed to that of 10% THF system showing a bisignate signal with positive and negative peaks at 482 and 550 nm respectively. The positive/negative pattern indicates the formation of left-handed helical aggregates which matches with the CD signal of the matured samples (Figure 3). The intensity of the signals increased with time but after 24 h no change was observed. Unlike the case of 10% THF, the time dependent FESEM study of 50% THF system showed (Figure 5D–F) fiber like structures at the initial stage (0 h) while both fiber as well as nano-rings were found after 12 h. After 24 h, the morphology was predominantly filled with nano-rings with occasional appearance of fibrous structures which was similar to that of the 72 h aged sample (Figure 1D).

**Figure 6:**
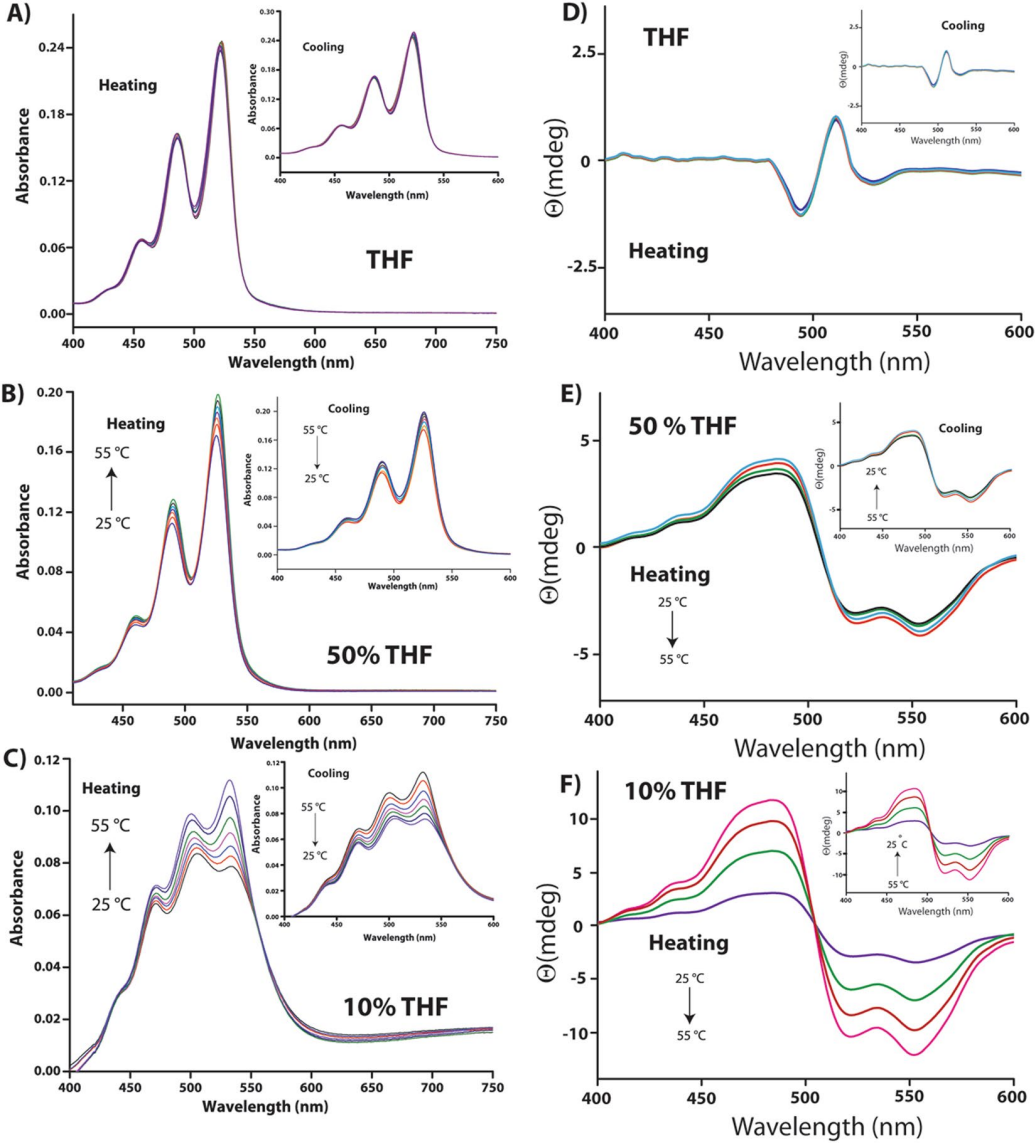
Temperature dependent absorption (**A**–**C**) and CD (**D**–**F**) spectra of 10 μM solutions of **P-1 **in different THF-water compositions.

To further consolidate the kinetic and thermodynamic control over the self-assembly of **P-1** in different solvent systems, temperature dependence of the absorption and CD spectra were recorded (Figure 6). Absorption and CD spectra recorded for **P-1 **in THF between 25 and 55 °C (at a rate of 1 °C min^−1^) showed no noticeable change during the heating and cooling cycles supporting the kinetic control over the assembly and the assembly is thermodynamically stable. Notably, in the case of 10% THF, both absorption and CD signals showed significant changes as a function of temperature. The absorption increased with increase in temperature followed by a reversal of the A_0–0_ and A_0–1_ ratio. The mole fraction of aggregate at each temperature (α_agg_(*T*)) estimated for both heating and cooling process showed no significant change in the curve (Figure S22, SI). Moreover changing the rate of heating cooling (from 1 to 0.1 °C min^−1^) also did not impart any change in these pattern. In case of CD signals, the intensity decreased with temperature as can be seen in Fig. 6. On cooling, both the spectral features restored back to their initial positions. The dependence of both absorption and CD signals as well as the (α_agg_(*T*)) values on temperature strongly support the thermodynamic nature of the assembly^43–45^. In the case of 50% THF, somewhat mixed responses were obtained. Both, absorption and CD signals showed slight changes as the absorption increases with temperature while the CD intensity decreased. However, the observed changes were much lower compared to that of the case of 10% THF system. The minor changes observed in this case may presumably due to the fact that both thermodynamic and kinetic factors control the assembly in this case which supports the appearance of mixed morphology in this case.

**Figure 7:**
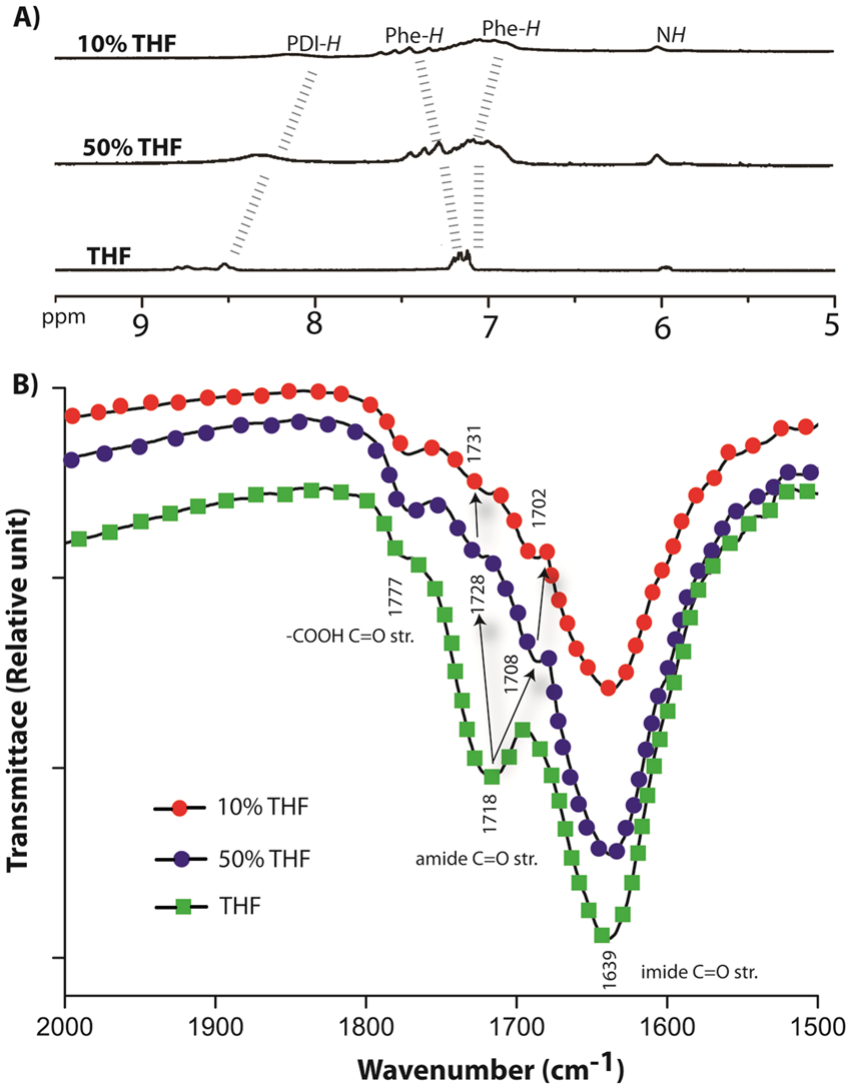
(**A**) ^1^H NMR (aromatic region) spectra and (**B**) IR (carbonyl region) of **P-1** (10 μM) from different THF-water compositions.

To further elucidate the self-assembly mechanism, ^1^H NMR and IR spectra of the matured samples were recorded. In deuterated THF (THF-*d*_8_), the aromatic and the amide protons appeared as sharp peaks which broadened considerably in presence of D_2_O (Figure 7A). In 50% THF-*d*_8_, a clear up-field shift of the aromatic protons arising from PDI core was observed. Interestingly, the phenylalanine protons showed two broad signals, one down-field shifted while the other slightly up-field shifted. With further increase in D_2_O, i.e. 10% THF-*d*_8_, these two sets of protons were further separated as one of them moved to higher chemical shift value while the other showed up-field shifts. Though it is possible that these splitted signals arise from two different “Phe” protons on each sides of the PDI core, the close proximity and similar chemical equivalence of these two “Phe” groups (as they appear at the same chemical shift values in case of THF-*d*_8_) make this possibility very unlikely. On the other hand, two different signals may arise from the “Phe” protons of two different sides of the PDI core if the environments are different around these two sides. As no conclusion could be drawn from the NMR study, IR spectra were recorded for these samples. As shown in Fig. 7B, the sample from THF showed three clear carbonyl stretching frequencies at 1639 cm^−1^ (imide), 1718 cm^−1^ (amide) and 1777 cm^−1^ (carboxylic acid)^34, 46^. As the water content enhanced, not only the intensity for the amide band decreased dramatically, the signal split in to two. The new signals arising from the amide bonds appear at 1708 cm^−1^ and 1728 cm^−1^ in case of 50% THF. In 10% THF, further shifts were observed and the bands appeared at 1702 and 1731 cm^−1^. As there is only one amide carbonyl group on each sides of PDI core, the splitting of the amide signal signifies they are under different environments. This phenomenon indicates that in 10% THF, two sides of the aggregated PDI cores are not equivalent which also explains the splitting of “Phe” protons in NMR study.

**Figure 8:**
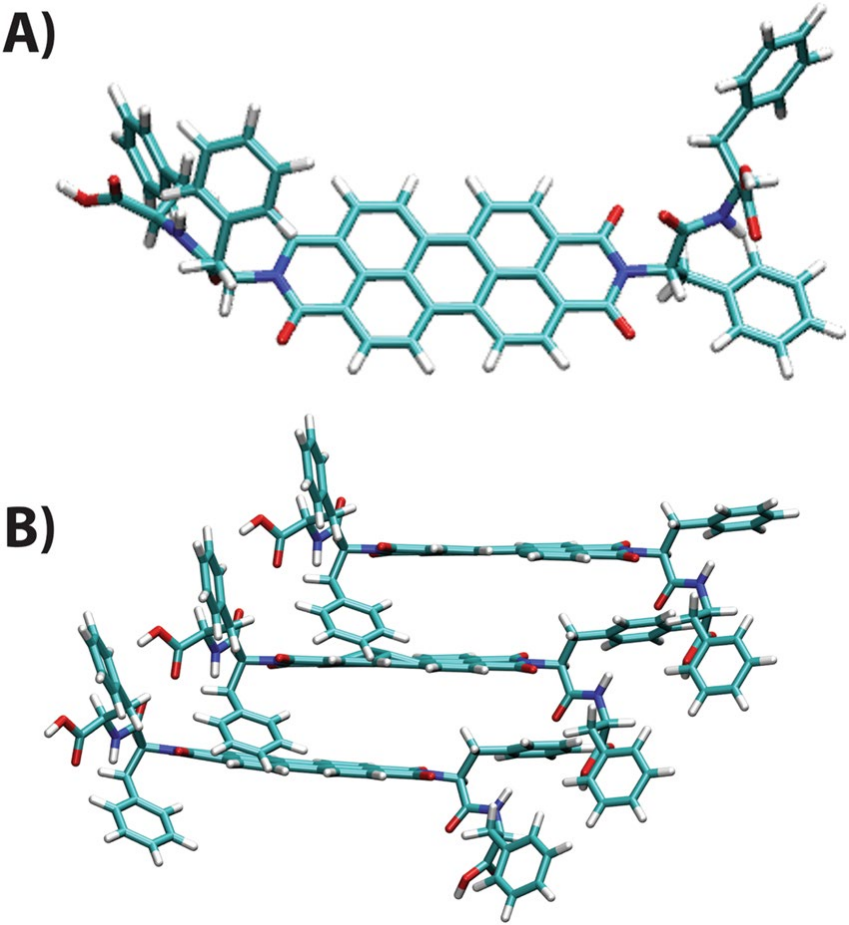
(**A**) Optimized geometry of the monomer from DFT calculations, (**B**) Left handed stacking arrangement of **P-1** molecule from DFTB calculations.

**Figure 9:**
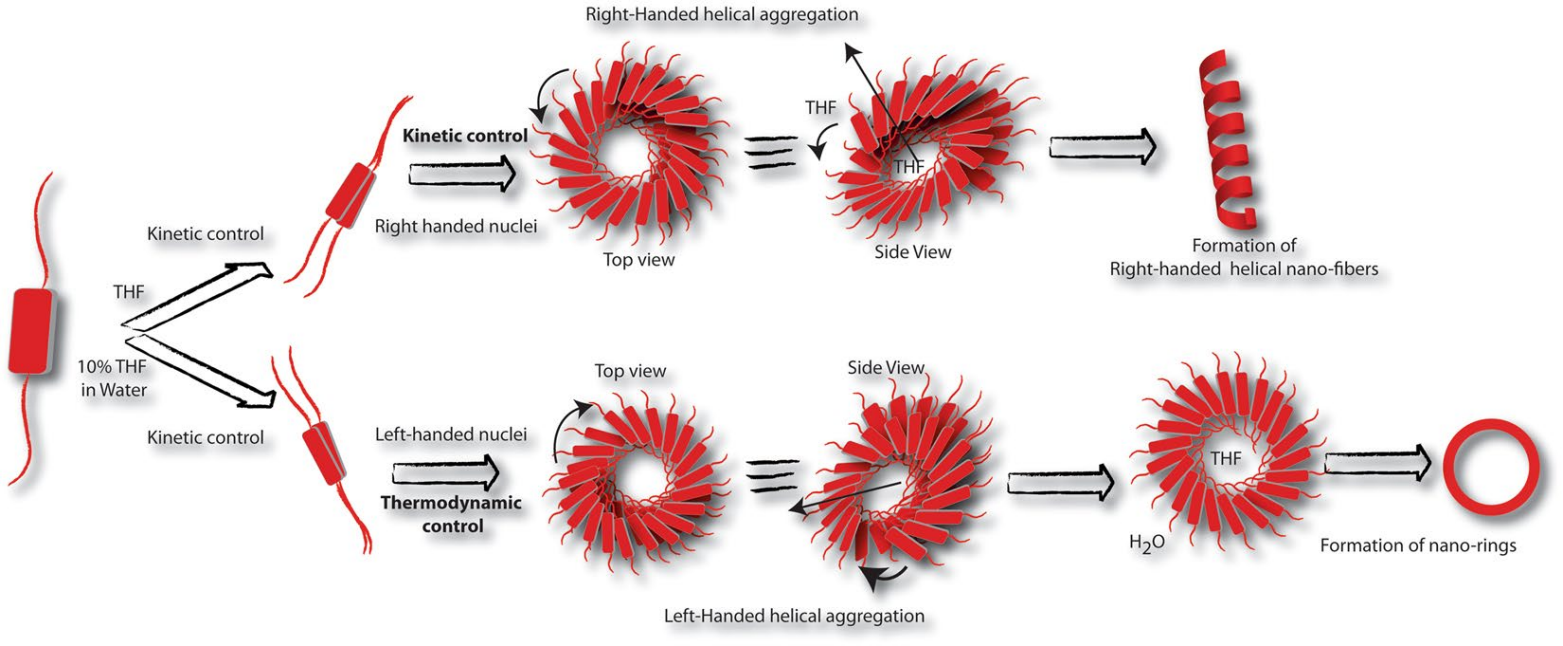
Schematic presentation of the thermodynamic and kinetic control of the self-assembly process of **P-1** in different solvent composition to show the formation mechanism for helical nano-fibers and nano-rings.

DFT calculations were performed to find out the energetically favorable mechanism of the self-assembly. Three initial configurations of PDIs are chosen based on relative orientations of “PhePhe” units (Figure S23, SI). These are geometry optimized (Figure S23, SI) to obtain the energetically most favorable structure. The most stable configuration forms the nucleus of the aggregates. Figure 8 shows how the optimized stable geometries can be stacked to form helical aggregates as proposed in Figure 9. The helical aggregate can form a nano-ring due to the attractive depletion energy of the expelled water from the core (where the THF molecules reside) which can be accommodated within the bulk-water near the “PhePhe” groups at outer rim of the nano-rings. Thus the inner and outer “PhePhe” groups are differentially solubilized in THF and bulk water respectively to provide an energetically favorable condition.

Based on the presented data and theoretical calculations, it is now possible to summarize the detailed mechanism of the self-assembly processes involved. **P-1** is sparingly soluble in water (<0.1 μM) but shows moderate solubility in THF (~0.1 mM) and thus THF can be termed as a good solvent and likewise, water as poor solvent for **P-1**. However, even in THF, the molecules form kinetically controlled aggregate. The molecules, owing to the presence of chiral residues, form right-handed helical nano-fibers (Figure 9). In presence of a poor solvent like water, the self-assembly occurs dominantly in a thermodynamically controlled fashion. In this case (10% THF), the nucleation of aggregates at the initial stage is a kinetically controlled process and leads to a left-handed helical nuclei. The nuclei further grow with time and the growth of the nuclei is thermodynamically driven. Following the kinetically controlled nucleation, owing to the ‘sergeant and soldiers rule’, the helicity of the assembly remained left-handed^47^. As the nuclei grow, in case of 10% THF, the THF molecules presumably tend to form an inner core where the “PhePhe” units of one side of the stack get easily solubilized. Being the poor solvent as well as more in number, water molecules tend to come out of this core region, keeping THF within the core in order to increase entropy where depletion energy plays a role. Therefore, the solubilization of the “PhePhe” units are different in the outer and inner rims of the nano-rings. This explains the presence of two types of Phenyl groups as well as two different amide signals in IR spectra. This differential solubility in turn creates a critical hydro-dynamic radius to chain length which does not allow the chain to grow along the long axis but forms the ring.

As mentioned in the introduction, the semiconducting property of PDI based molecules is of immense importance owing to their applications in organic-electronics. However, only few studies has so far been reported to correlate the conducting property with the morphology of these self-assembled n-type semiconductors^48, 49^. In order to evaluate the relation between nano-structures with their conducting ability, the helical nano-fibers and nano-rings were subjected to Conducting – Atomic Force Microscopy (C-AFM).

**Figure 10:**
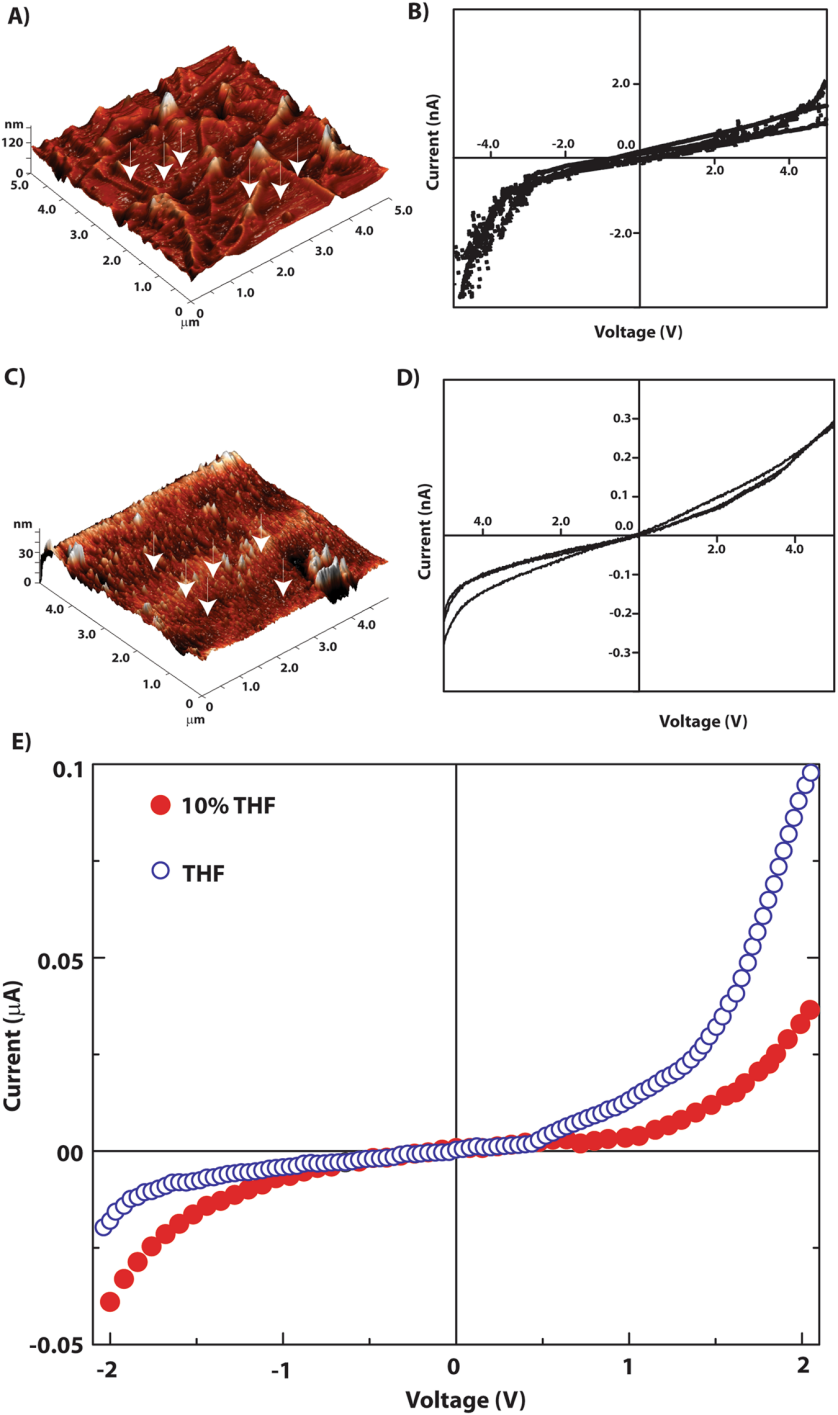
(**A**–**D**) C-AFM analyses of thin film of **P-1** (10 μM) prepared from THF (**A**,**B**) and 10% THF (**C**,**D**). (**A** and **C**) 3D topography of the nanofibers and nano-rings respectively; (**B** and **D**) I-V characteristics of nanofiber and nano-rings obtained from C-AFM. (**E**) Current-voltage characteristics of **P-1**. The thin films of **P-1** have been deposited from different solvents as the legends indicate.

The topography of the nanofibers and nano-rings has been observed in height profile of contact mode AFM images. For THF system, 3-dimensional images of nanofibers were obtained all over the surface (Figure 10A). The scanning resistance image (SRI, Figure S24, SI) shows higher current at the nano-fiber regions and dark points correspond to low current regions on the substrate. With the set point bias the maximum current recorded is around 150 pA. The current-voltage characteristics have been recorded at the probe points (points indicated by arrows, Figure 10). These probe points were carefully chosen so that the probe can land either on nano-fiber or on valley. For current-voltage characterization, a bias sweep from −5 V to +5 V has been applied and respective currents were recorded. These characteristics show quite higher current value, whereas an abrupt increase in current can be seen in negative bias after −4 V. The characteristics testify that the probed nanostructures are electronically active and semiconducting in nature. This is affirmed from the increasing conductivity and the nature of the characteristics – which signifies that there should be a band gap to facilitate charge flow through them.

A similar treatment with the sample from 10% THF system revealed the formation of nano-rings on the surface as can be seen from Fig. 10C–D. A higher valley, distinctive of the lower back-ground was observed which might apparently be formed by coalescing the nano-rings side-by-side. The SRI (Figure S24, SI) also confirms the same where this structure seems as a brighter patch. Though the nano-rings are formed by the aggregation of **P-1**, the conductivity is much lesser than that of the nanofibers as the maximum current recorded at set point condition is 30 pA. The lower conductivity is also observable from the current-voltage characteristics. These current-voltage characteristics from the bright section of the SRI have semiconducting nature too, however, they may have higher band gap. Due to this higher band gap, the work-function mismatch between tip and substrate is not noticeable in the current-voltage characteristics.

Though both the nanostructures are made up of same molecule, the constructional and size difference between them may have resulted higher effective band-gap for nano-rings. It is also plausible that interfaces between nano-rings restraint the charge transport through the nano-rings. The nano-fiber structures have advantage of percolating path of charge flow whereas charges possibly get localized within the confined structure of nano-rings. While for nano-fibers, charges transmitting transversely *i.e*. across the diameter, in case of planar nano-rings, charges are passing through the core of the molecules. Nevertheless, the current voltage characteristics of the nano-fibers and nano-rings reveal that the aggregation pattern and the morphology play vital role in the semiconducting nature of these materials.

For conventional current-voltage (*I*-*V*) characterization, we have carried out *I*-*V* measurements in thin film configuration. Thin films were deposited from THF or 10% THF-water by slow spin cast method. With the used concentration of **P-1** in these solvents, the expected thickness of the films could be ~200 nm. These films were sandwiched between two Al electrodes deposited *via* thermal evaporation. Figure 9E shows the *I-V* characteristics of thin films within a bias range of −2  to +2 V. From these measurements it is clearly seen that the thin film from the mixed solvent is semiconducting while that from THF shows current rectifying behavior. These electronic natures of two different types of nanostructures have been indicated in C-AFM study. Here, as we have electrodes of same metal on both sides of the film, the effect of built-in potential can be negated. Thus the current rectification behavior may be attributed to conformational changes which has different local energy levels^50^. It also must be noted that the charge conduction in nano-fiber network takes place through the delocalized PhePhe groups of the molecules. This is associated with organized hopping sites for charge carriers^51^. Specific orientation of molecules provides preferable direction of charge transmission which may cause the current rectification in nano-fiber network. A detailed investigation correlating the morphology and conducting property of such organic semiconductors is currently under progress.

## Conclusion

The mechanistic detail of the solvent controlled self-assembly of a peptide-PDI conjugate in the form of **P-1** has been revealed. The observed experimental results combined with theoretical calculations show that, in THF, **P-1** forms kinetically controlled right-handed helical nano-fibers. In presence of high percentage of water, *i.e*. in 10% THF, the initial nucleation is kinetically controlled and a left-handed helical nuclei is formed, which grows further in a thermo-dynamically controlled manner. A differential solubilization of the “PhePhe” motif on two sides of the fiber leads to the proper curvature which allow the formation of the nano-rings. C-AFM and *I-V* characterization studies of the morphologies show that the nano-fibers are much superior to the nano-rings in their semiconducting behavior. Thin film obtained from mixed solvent was found to be semiconducting while the film from THF showed current rectifying behavior. These observations reveal the critical role of self-organization mechanism towards their applicability and possibility of fine-tuning the semiconducting properties by modulating the morphology of aggregates.

## Materials and Methods

### General

All the chemicals and reagents used were obtained from Sigma-Aldrich (USA) and used without further purification. All solvents were procured from Merck, India and Spectrochem, India. To prepare samples, Milli-Q water with a conductivity of less than 2 μS cm^−1^ was used. ^1^H NMR, ^13^C NMR spectra were recorded on either a Bruker Ascend 600 MHz (Bruker, Coventry, UK) or an Oxford AS400 (Varian) spectrometer. ESI-MS spectra were recorded using a Q-Tof-Micro Quadrupole mass spectrometer (Micromass). MALDI-TOF was measured on a Bruker Autoflex Speed mass spectrometer.

### Synthesis

Syntheses and characterization of the compounds is given in the Supplementary information.

### Sample preparation

All the samples were prepared in Class-A volumetric flasks by weighing appropriate amount of the compound and dissolving in the corresponding premixed solvent mixtures. Unless otherwise mentioned, all the working solution were kept undisturbed for at 72 h prior to perform any experiment.

### NMR spectroscopy

^1^H, ^13^C spectra were recorded in deuterated Chloroform (CDCl_3_), dimethyl sulfoxide (DMSO-*d*_6_), Tetrahydrofuran (THF-*d*_8_) and heavy water (D_2_O) at 298 K and processed with standard 1D software. Whenever necessary, the NMR spectra were appropriately water suppressed for clarity.

### Absorption and fluorescence spectroscopy

Absorption and fluorescence spectra were recorded on a Lambda 750 (Perkin Elmer) and a Cary Eclipse (Agilent) spectrophotometers respectively.

### Circular Dichroism (CD) spectroscopy

The CD spectra of all the samples were recorded by using a 1.2 mL quartz cuvette of 0.5-mm path length with a Jasco J-1500 spectropolarimeter at RT. Spectra were collected at a scan rate 200 nmSec^−1^ and 2-nm bandwidth from 190 to 600 nm with three-times scans for averaging. Before running the sample the solvent was run to correct baseline.

### Fourier transformed infrared spectroscopy (FTIR)

The 72 h aged solutions of **P-1** were freeze-dried. KBr pellets were prepared by mixing the freeze-dried samples and oven dried KBr. The spectra were recorded on a Nicolet is 10 spectrometer. The baseline was subtracted from the obtained absorbance intensity in each case.

### FESEM

FESEM samples were prepared by casting a drop of dilute solution on a silicon wafer and air dried for at least 1 day. The samples with desired solvent compositions were prepared and incubated for at least 72 days before casting on the silicon wafer. For time dependent morphology analyses, samples were drop casted at the specified times. FESEM images were taken using a SIGMA ZEISS microscope.

### TEM

A 5 μL very dilute solution of the sample was casted on the carbon coated copper grid (300 mesh Cu grid with thick carbon film from Pacific Grid Tech, USA) and allowed to air dry for 2 minutes and then the excess sample was bloated with a tissue paper. The grid was then air dried for 1 day. The samples with desired solvent compositions were prepared and incubated for at least 3 days before casting on the grid. TEM images were taken in JEOL JEM-2100 microscopes.

### Calculation of α_agg_(*T*)

The mole fraction of aggregate (α_agg_(*T*)) was estimated by using Equation^52^,

$${{\rm{\alpha }}}_{{\rm{agg}}}(T)\approx (A(T)-{A}_{{\rm{mon}}})/({A}_{{\rm{agg}}}-{A}_{{\rm{mon}}})$$

where, α_agg_(*T*) is the mole fraction of aggregate at temperature *T*, and *A*_mon_, *A*(*T*), and *A*_agg_ are the absorbance at 517 nm for the monomer, the solution at temperature at temperature *T*, and the pure aggregate solutions respectively.

### Conductive AFM

The C-AFM had been carried out with drop casted film on doped Si substrates. The films were dried in closed ambient condition for a day and then dusted off by slow N_2_ gas blowing. C-AFM characteristics were measured using a NT-MDT NTEGRA Aura Atomic Force Microscope. A gold coated highly conductive tip was used to probe at 10 kHz. The C-AFM has a gold coated tip as biasing electrode. Doped Si substrate is highly conducting and could serve the purpose of ground electrode. The set point bias and current were fixed at 300 mV and 0.1 nA respectively.

### Electrical characterization

Thin films have been prepared from solutions of **P-1** in appropriate solvent(s). Spin-coating at 1000 rpm has been applied to fabricate the thin films on Aluminum coated glass substrates. Top electrode is thermally evaporated Al on the thin films. I-V characteristics were taken by Keithley 2450 source measure unit.

### Computational details for geometry optimization of **P-1**

Geometry optimization of **P-1** was performed using density functional theory (DFT) provided by DMol3 code^53, 54^ using Gaussian09 software^55^. Three initial structures (Figure S20A–C, SI) were optimized using semi-empirical method AM1^56^ with zero differential overlap (ZDO) basis sets. The optimized geometries obtained from semi-empirical calculations were further optimized using UB3LYP ^57–59^ and 6–31G9(d) basis sets which are shown in Figure S20D–F (SI). On comparing the energies of these con-figurations, geometry (D) is found to be energetically favorable (shown in Fig. 8A) than the other two. Since electronic structure calculation of PDI dimer is intractable using DFT due to its complex large structure, density-functional tight binding (DFTB)^60, 61^ method is used to calculate the energy of the dimer with different rotation angles. The calculations in DFTB are performed within DFT framework using either Slater-Koster integrals or from reference structures from DFT calculations. The binding energies of the dimers are calculated from the difference in energies between the dimer and the two monomers. The dimer with a rotation angle −55.20 degree is found to have the lowest binding energy (−10.81 kcal/mol) in compare to the dimers with other angles of rotation. This conformation can lead to a left handed stacking through significant π–π interactions. Moreover, inter-motif OH··O distances between carboxyl group of the side chain and amide oxygen of PDI are within a hydrogen bond distance. Thus, this configuration of the dimer can act as a motif to the thermodynamically stable nano-rings. (Figure 8B) shows the stacking arrangement of **P-1** molecules along z axis in order to form the nucleus of the aggregates. This result matches well with our experimental findings where the **P-1 **molecules stack in the thermodynamically controlled structure to form nano-rings.

## Electronic supplementary material

Supplementary Information 
