# Supplementary material for: Solvent Assisted Tuning of Morphology of a Peptide-Perylenediimide Conjugate: Helical Fibers to Nano-Rings and their Differential Semiconductivity
Source: Sci Rep. 2017 Aug 25;7:9485. doi: 10.1038/s41598-017-09730-z (PMC5573372; doi:10.1038/s41598-017-09730-z)
Supplement: Supplementary file 1 — Supplementary Information [file 41598_2017_9730_MOESM1_ESM.pdf]

Supplementary information

Solvent Assisted Tuning of Morphology of a Peptide-Perylenediimide Conjugate: Helical Fibers to Nano-Rings and their Differential Semiconductivity

Sahnawaz Ahmed, Bapan Pramanik, K. N. Amba Sankar, Abhinav Srivastava, Nilotpal Singha, Payel Dowari, Arpita Srivastava, Kallol Mohanta, Ananya Debnath, and Debapratim Das[*]

Synthesis:

P-1and D-**P-1**were prepared following **Scheme S1** starting from L- and D- phenylalanine respectively.

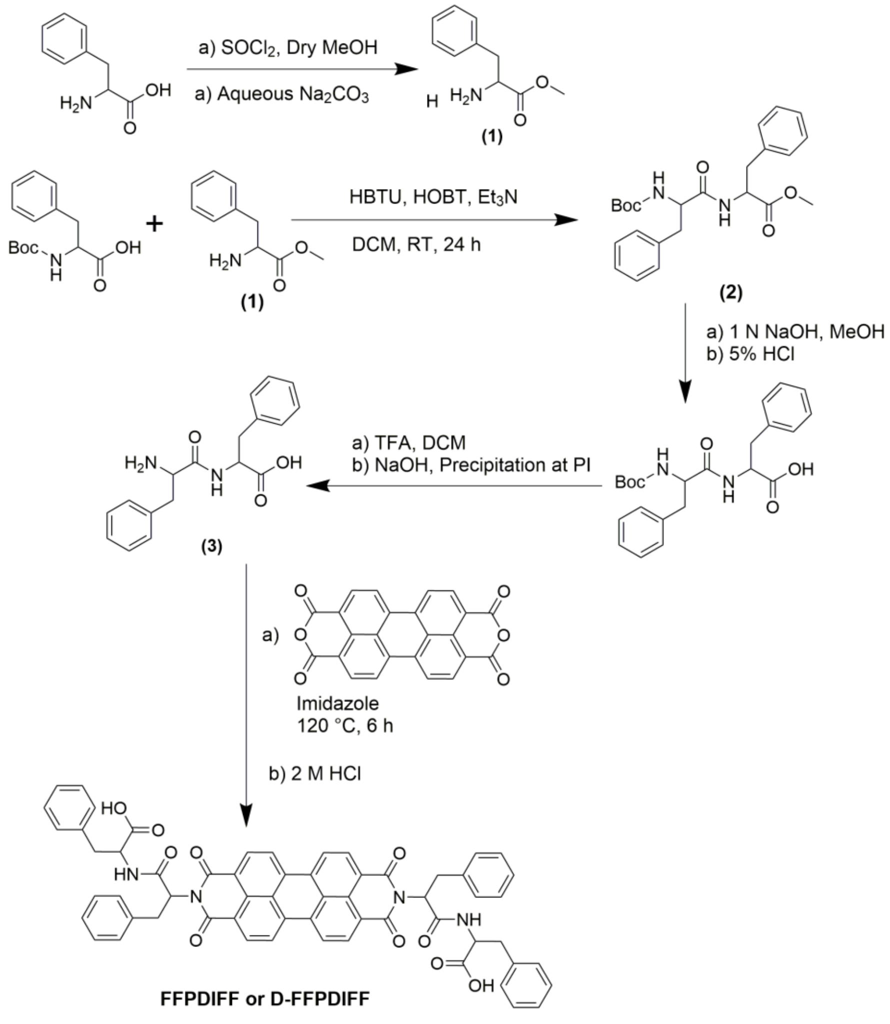

Scheme S1. Synthetic route for compound **P-1**and D-**P-1**.

Methylphenylananinate (1): 1 g (6.06 mmol, 1 equiv.) phenylalanine was dissolved in 15 ml dry methanol and placed in an ice bath. To this solution, 3 ml of freshly distilled thionyl chloride was added dropwise with stirring over a period of 15 mins. The reaction mixture was then allowed to come to room temperature and stirred overnight. After that, solvents were removed under reduced pressure and saturated aqueous Na_2CO_3 solution was added to neutralize. The methyl ester of phenylalanine was extracted with dichloromethane (DCM) and washed with brine. The organic layer was dried over anhydrous Na_2SO_4 followed by removal of the solvent under reduced pressure to get an oily product with 94% yield (0.95 g, 5.6 mmol). ^1H NMR ($CDCl_3$, 600 MHz): δ (ppm) = 7.36 – 7.22 (m, 5H), 3.79 – 3.77 (t, *J* = 6.0 Hz, 1H), 3.75 (s, 3H), 3.15 – 3.11 (dd, 1H), 2.92 – 2.87 (dd, 1H). ^{13}C NMR ($CDCl_3$, 150 MHz): δ (ppm) = 167.13, 135.03, 129.77, 129.21, 127.77, 55.60, 50.83, 39.72. Mass (ESI-MS): *m/z* calcd. for $C_{10}H_{13}NO_2$: 179.0946, found 180.1026 $[M + H]^+$.

Methyl (tert-butoxycorbonyl)phenylalanine phenylalaninate (2): 0.895 g (5 mmol, 1equiv. compound **1** was coupled with 1.59 g (6 mmol, 1.2 equiv.) Boc-Phe-OH in DCM using 2.274 g (6 mmol, 1.2 equiv.) HBTU, 1.214 g (12 mmol, equiv.) trimethylamine and 0.81 g (6 mmol,1.2 equiv.) HOBT for 24 h. The reaction mixture was then washed with brine and the organic layer was dried over anhydrous Na_2SO_4 and filtered. The solvent was removed under reduced pressure to get a white crude product. The crude mixture was subjected to column chromatography on a 60-120 mesh silica gel column using ethylacetate/hexane as the eluent to yield 1.64 g (3.85 mmol) of the title compound. Yield: 77%. ^1H NMR ($CDCl_3$, 600 MHz): δ (ppm) = 7.30-7.27 (t, *J* = 9.0 Hz, 2H), 7.24-7.22 (m, 4H), 7.19-7.18 (d, *J* = 6.0 Hz, 2H), 6.98-6.97 (d, *J* = 6.0 Hz, 2H), 6.27 (br, 1H), 4.94 (br, 1H), 4.78 (br, 1H), 4.3 (br, 1H), 3.67 (s, 3H), 3.06-3.01 (m, 4H), 1.4 (s, 9H). ^{13}C NMR ($CDCl_3$, 150 MHz): δ (ppm) = 171.42, 170.95, 155.30, 136.61, 135.72, 129.34, 129.21, 128.53, 128.48, 127.03, 126.84, 79.98, 55.60, 53.29, 52.17, 38.36, 37.93, 28.22. Mass (ESI-MS): *m/z* calcd. for $C_{24}H_{30}N_2O_5$: 426.2155, found 427.2253 $[M+H]^+$.

Phenylalaninephenylalanine (3): Compound **2** was subjected to ester hydrolysis. For that, 1.5 g (3.5 mmol) of **2** was dissolved in methanol and to it 15 ml of 1 N NaOH solution was added and refluxed with stirring overnight. Methanol was then evaporated and 5% HCl was added to bring the system to pH 2 to get a white precipitate. The white product was extracted with DCM and dried over anhydrous Na_2SO_4, filtered and the filtrate was

concentrated under reduced pressure. It was then subjected to Boc-deprotection by a 15 ml of 2:1 trifluoroacetic acid (TFA) – DCM mixture. After 2 h of stirring, solvents were removed under reduced pressure. To the residue, dry diethyl ether was added, a white ppt. appeared which was washed with dry ether repeatedly to get a white solid as trifluoroacetate salt of diphenylalanine. The solid was dissolved in water and the pH was adjusted to it's isoelectric point (PI) to get free diphenylalanine (**3**) with overall yield 80% (0.88 g, 2.82 mmol). ^{1}H NMR (DMSO-d_6, 600 MHz): δ (ppm) = 7.17-7.04 (m, 10H), 4.45-4.43(t, J = 6.0 Hz, 1H), 3.76-3.74(t, J = 6.0 Hz, 1H), 3.06-3.01 (m, 1H), 2.96-2.84 (m, 2H), 2.64-2.55 (m, 1H). ^{13}C NMR (DMSO-d_6, 150 MHz): δ (ppm) = 172.64, 157.70, 137.64, 136.42, 129.40, 129.27, 128.27, 128.09, 126.61, 126.35, 54.75, 53.61, 37.28, 37.00. Mass (ESI-MS): m/z calcd. For $C_{18}H_{20}N_2O_3$: 312.1474, found 313.1591 $[M+H]^+$.

P-1 or D-P-1: 0.3 g (0.77 mmol, 1 eqv. perylenetetracarboxylic dianhydride (PTCDA), 0.5 g (1.62 mmol, 2.1 equiv.) compound **3** and imidazole 2.08 g (30.8 mmol, 40 equiv.) were mixed in a round bottom flask. Argon was purged for 15 minutes before heating the mixture at 120 °C for 6 h. The reaction mixture was cooled to 90 °C. Water was then added to the reaction mixture with the protection of argon and the reaction was continued for another 1 h. The reaction was cooled to room temperature and it was filtered to remove the trace amount of unreacted PTCDA. The solution was then acidified with 2M HCl solution to pH 3, the precipitate was collected by centrifugation and washed thoroughly with water until the filtrate become neutral. The solid was suspended in 20 ml water, frozen in liquid nitrogen and lyophilized to get the desired final compound with 61% yield (0.46 g, 0.47 mmol).^{1}H NMR (DMSO-d_6, 400 MHz): δ (ppm) = 8.45-8.25 (m, 8H), 7.28-7.08 (m, 20H), 5.84-5.82 (t, J = 4.0 Hz, 2H), 4.46 (s, 2H), 3.70-3.67 (dd, 2H), 3.09-3.12 (dd, 2H), 2.94-2.92 (dd, 2H), 2.84-2.81 (m, 2H). ^{13}C NMR (DMSO-d_6, 150 MHz): δ (ppm) = 173.26, 168.85, 162.51, 138.04, 133.94, 131.34, 129.47, 129.19, 128.45, 128.30, 126.59, 125.56, 123.96, 122.90, 55.26, 54.45, 36.40, 34.19. Mass (MALDI-TOF, DHB matrix): m/z calcd. for $C_{60}H_{44}N_4O_{10}$: 980.3057, found 980.8560 $[M]^+$.

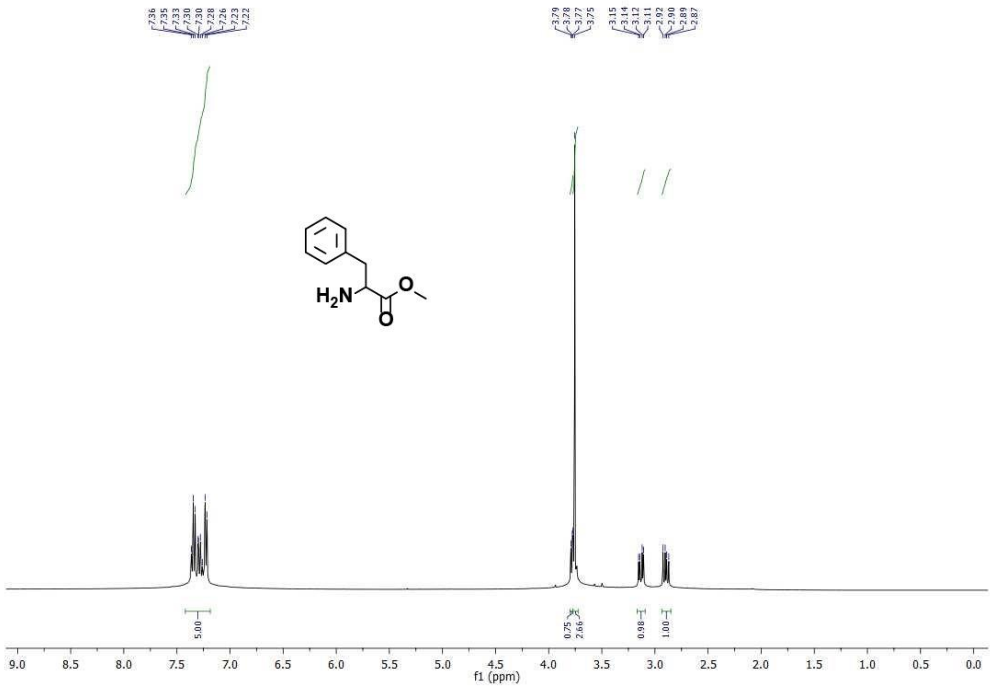

Figure S1. ^{1}H NMR Compound **1**

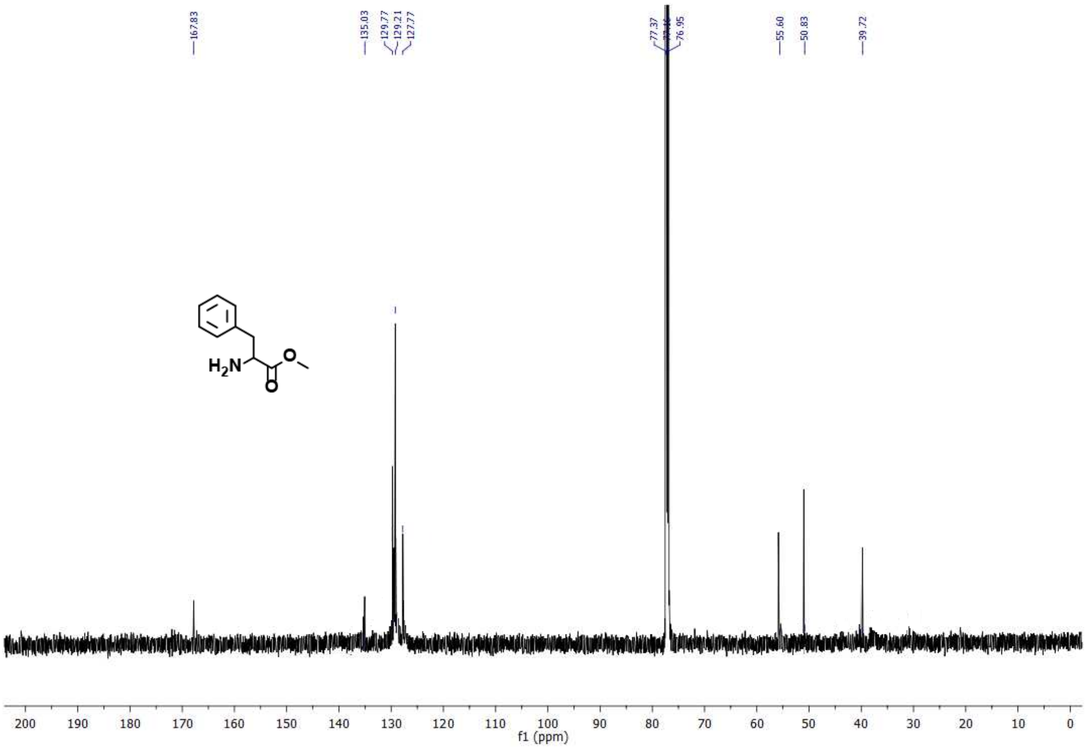

Figure S2. ^{13}C NMR Compound **1**

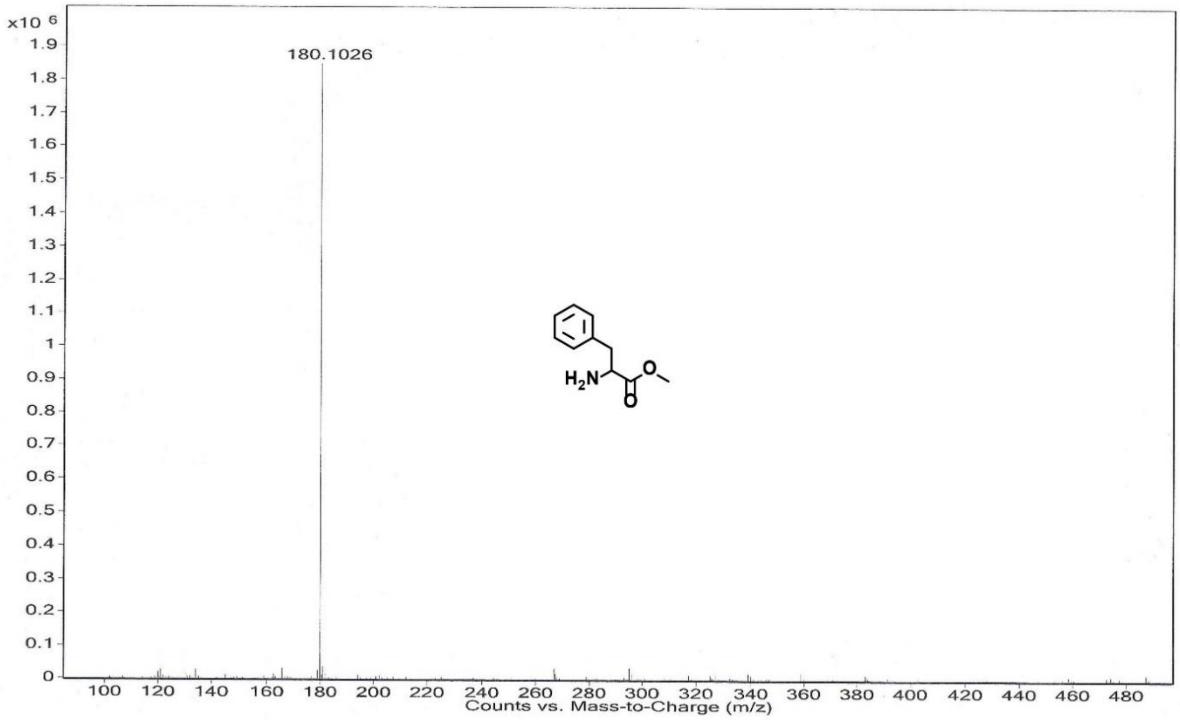

Figure S3. ESI-Mass of Compound **1**

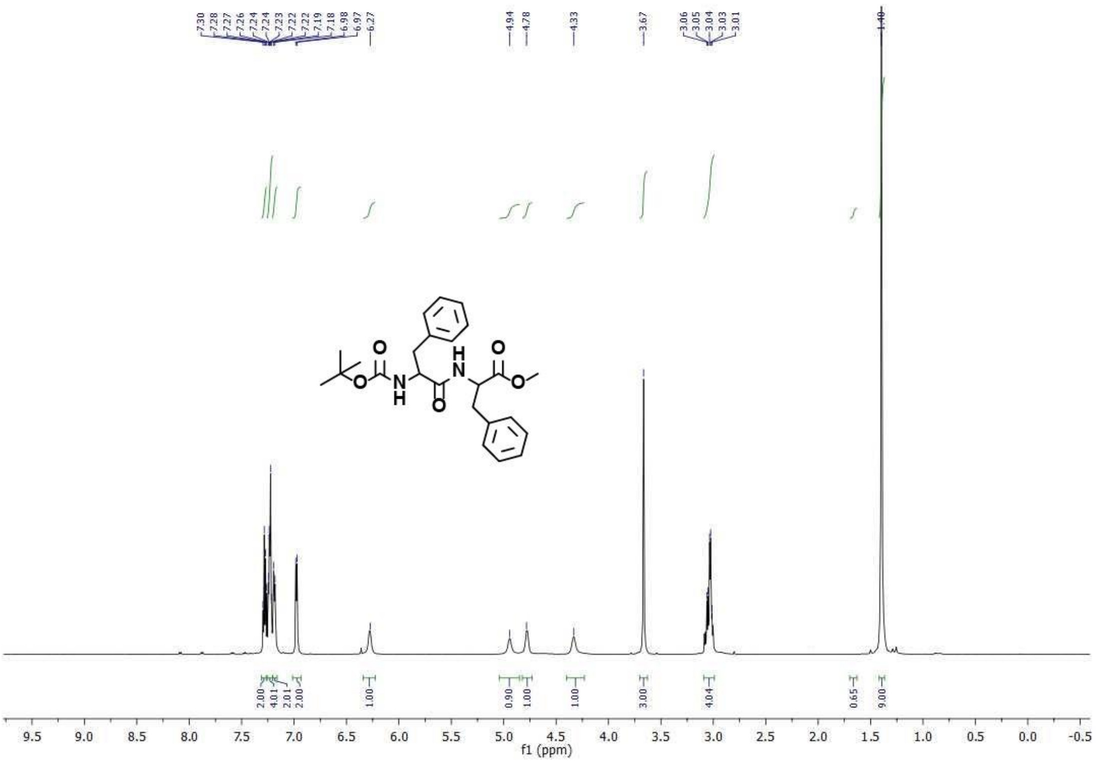

Figure S4. ^{1}H NMR Compound **2**

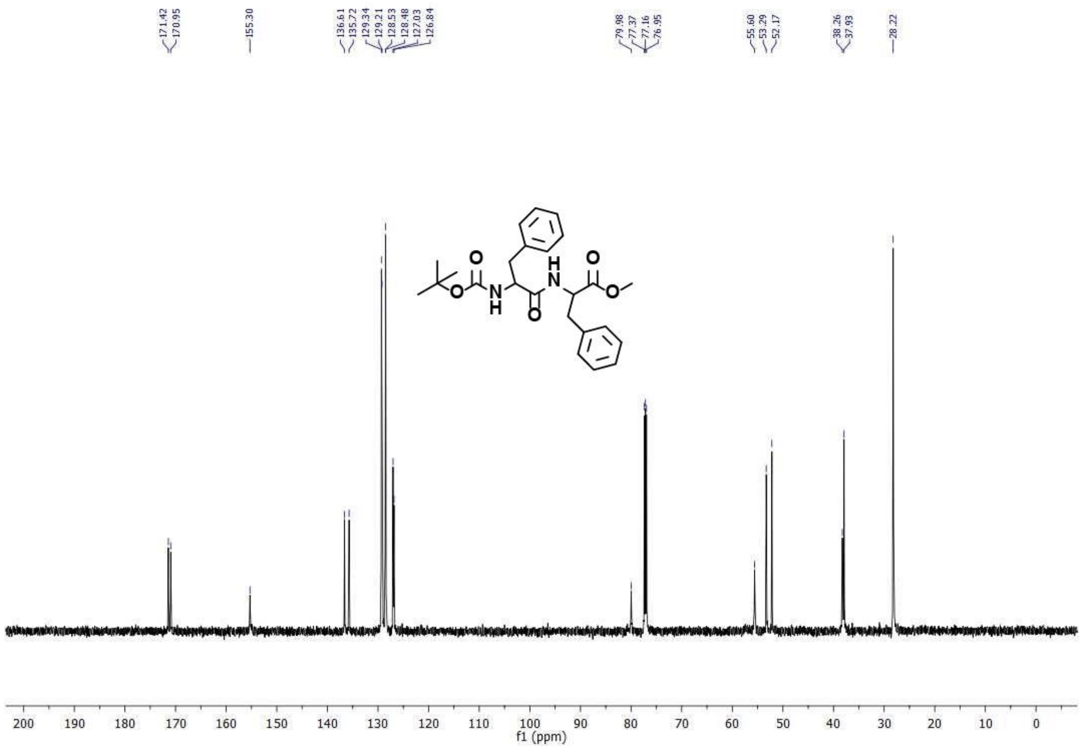

Figure S5. ^{13}C NMR Compound **2**

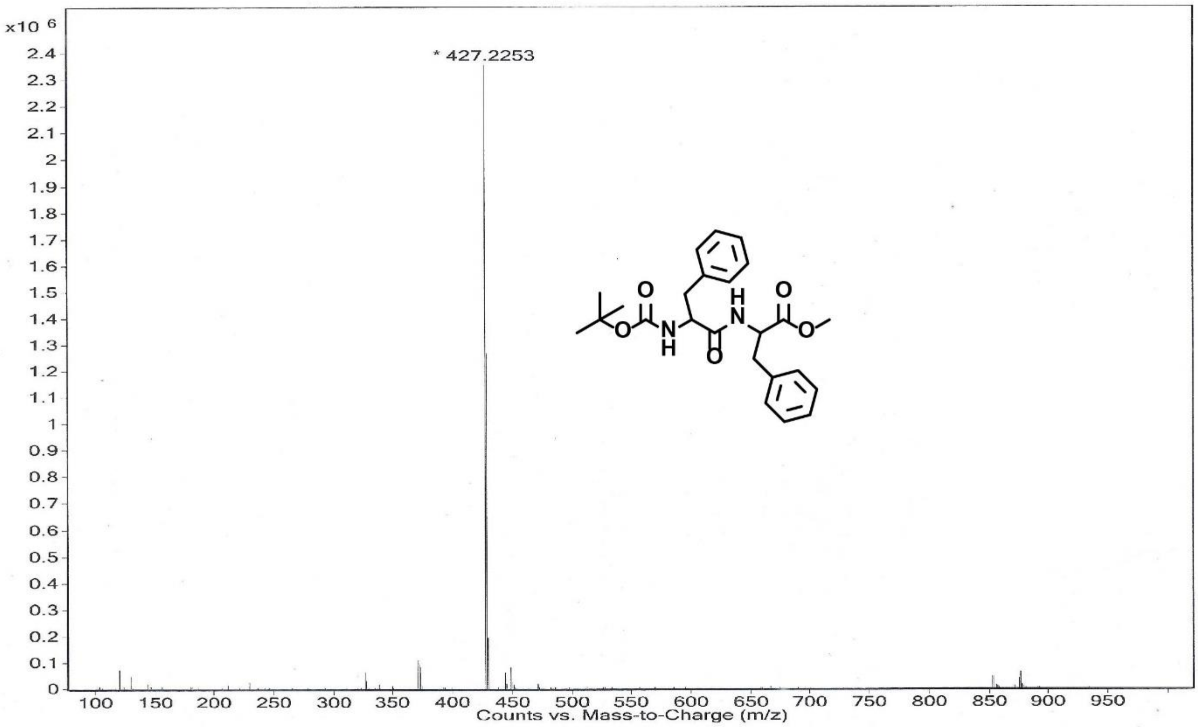

Figure S6. SI-Mass of Compound **2**

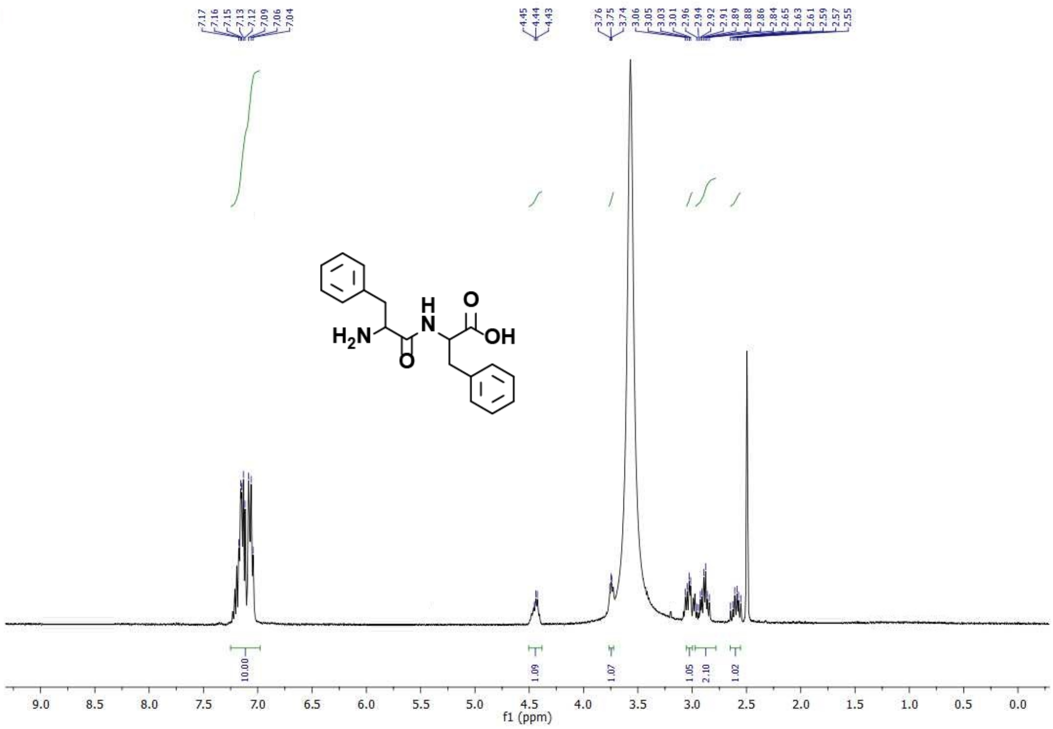

Figure S7. ^{1}H NMR Compound **3**.

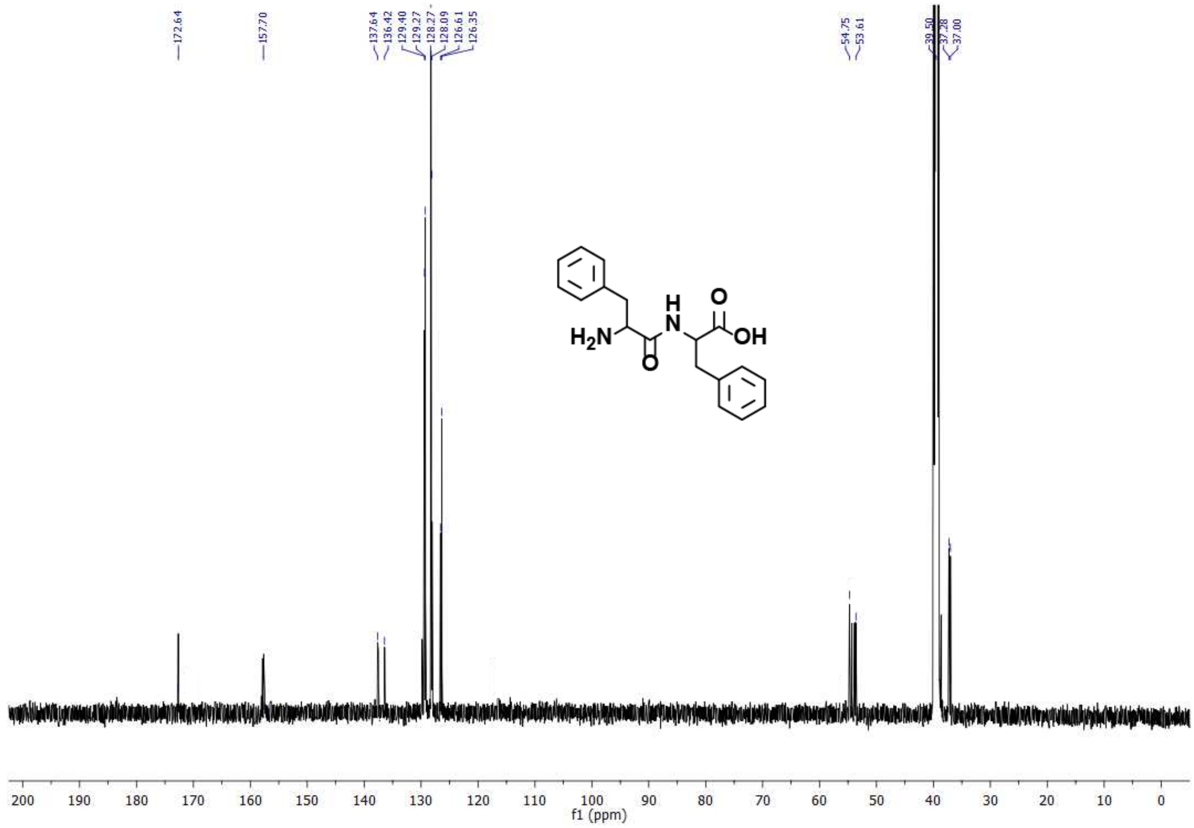

Figure S8. ^{1}H NMR Compound **3**.

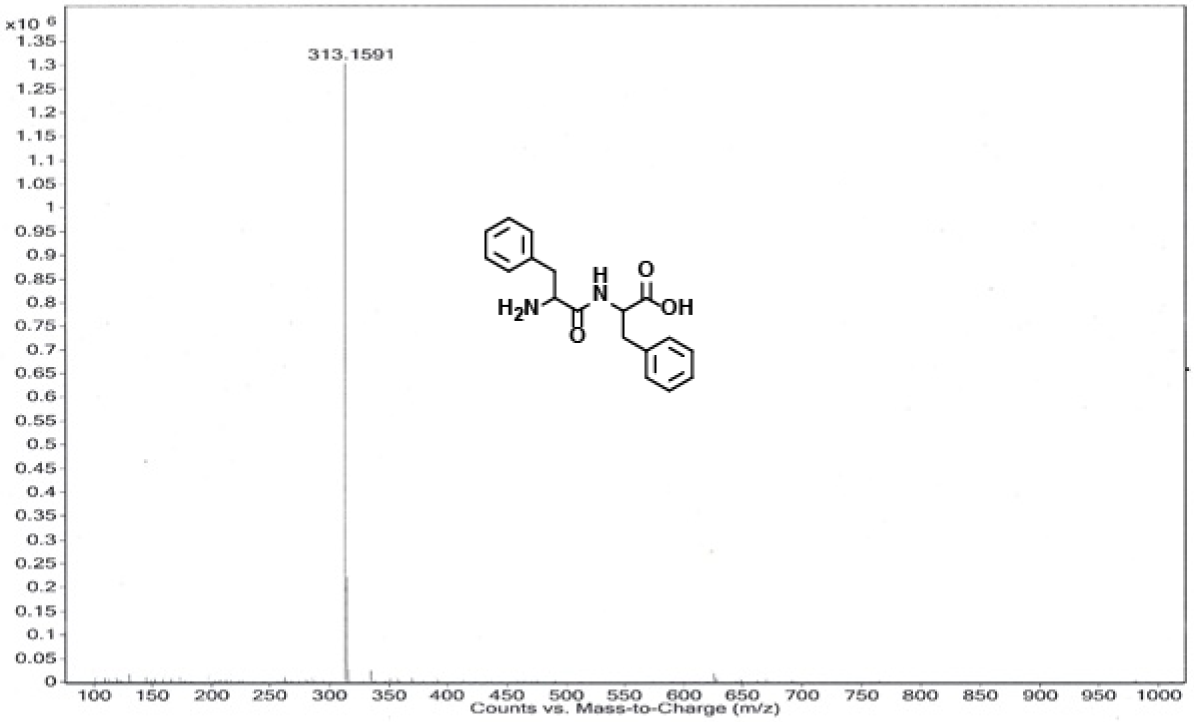

Figure S9. ESI-Mass of Compound **3**.

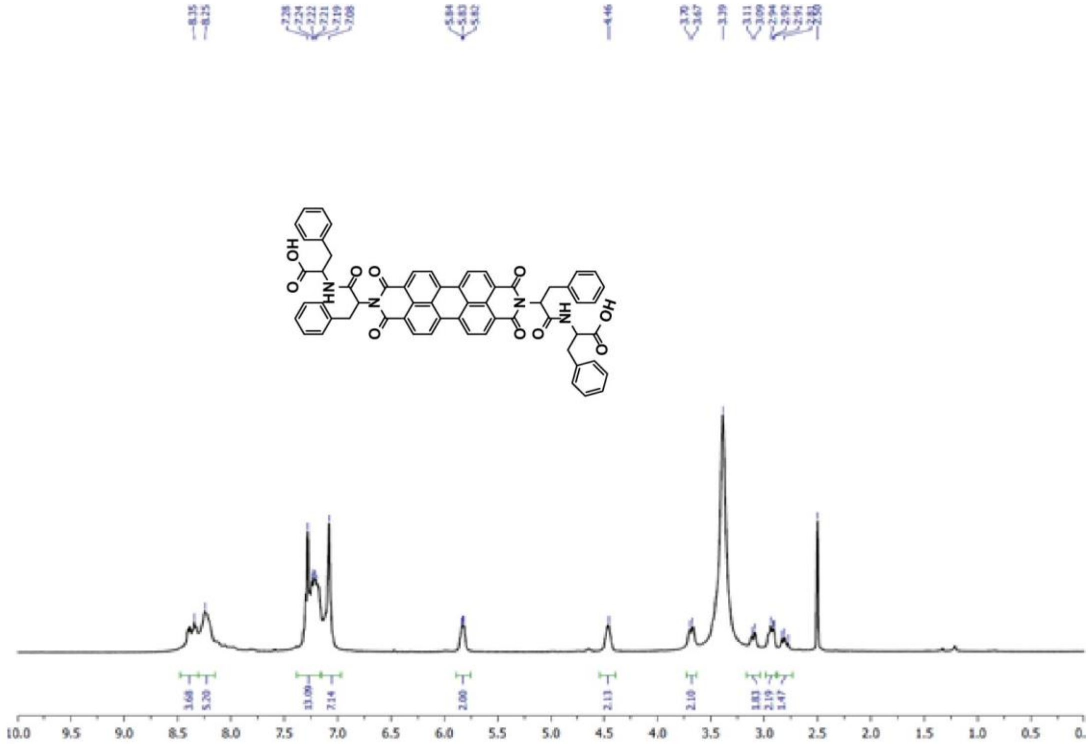

Figure S10. ^{1}H NMR Compound **P-1**.

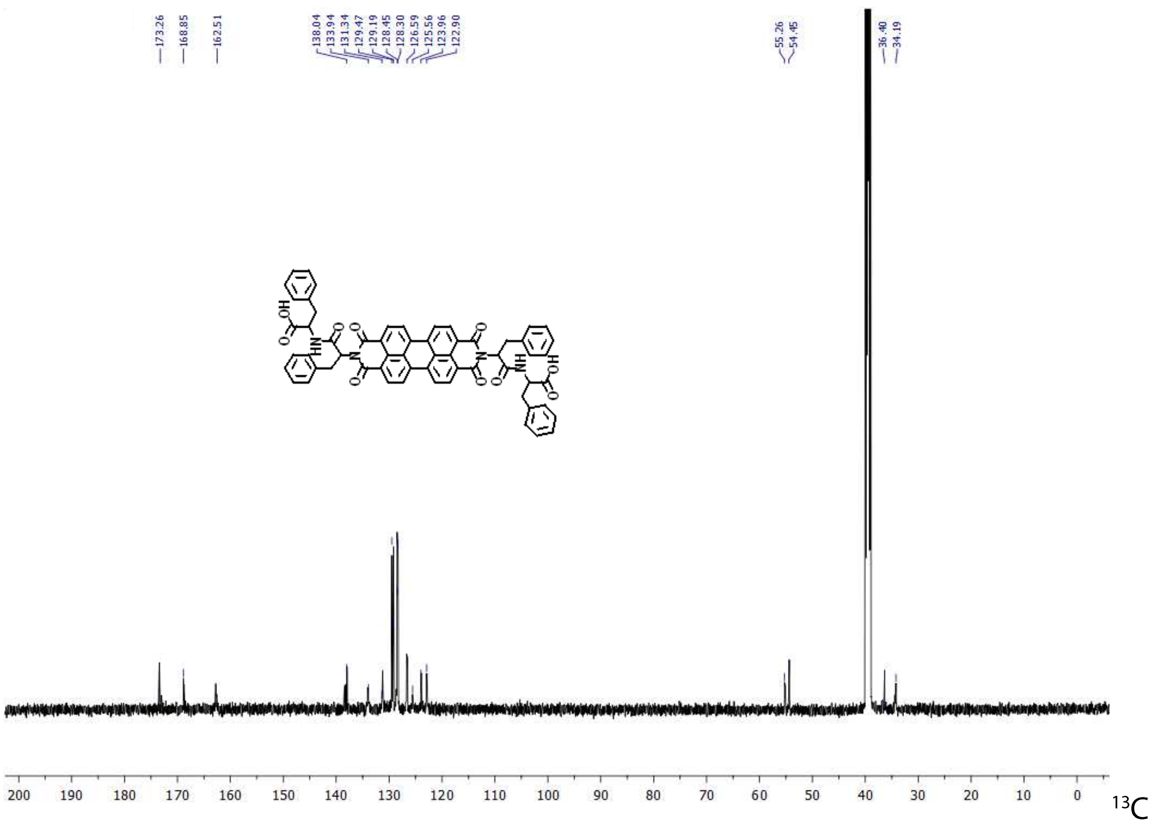

Figure S11. ^{13}C NMR Compound **P-1**.

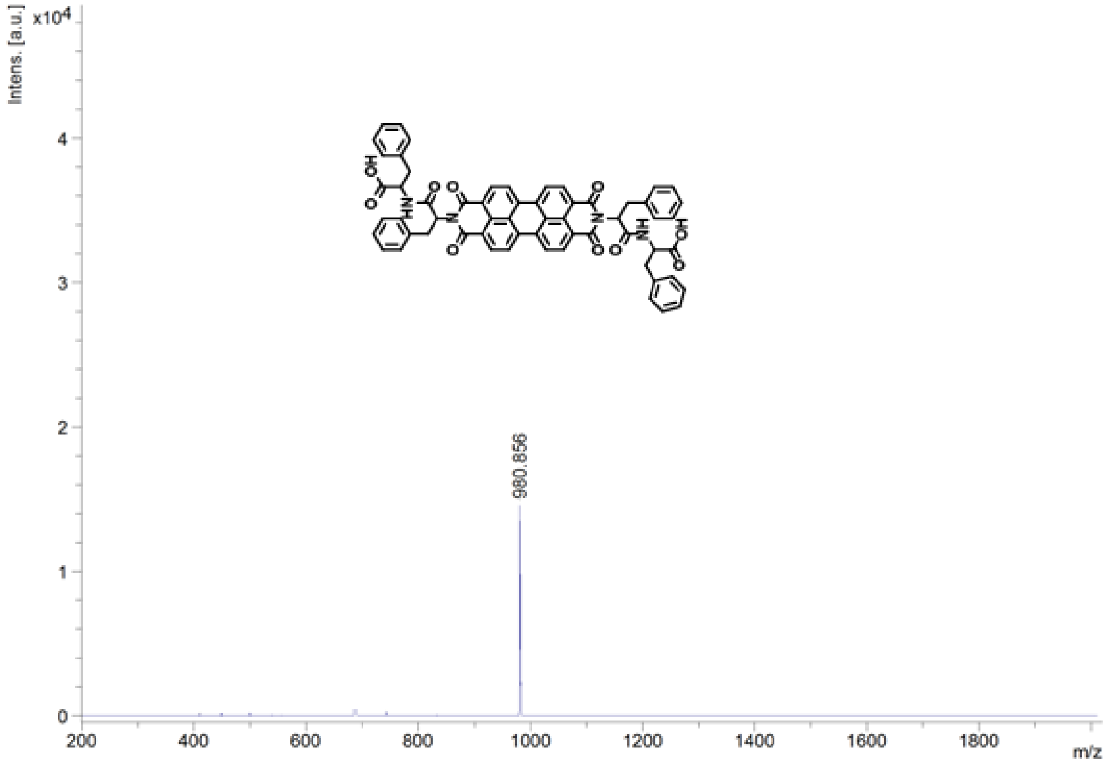

Figure S12. MALDI-TOF Compound **P-1**.

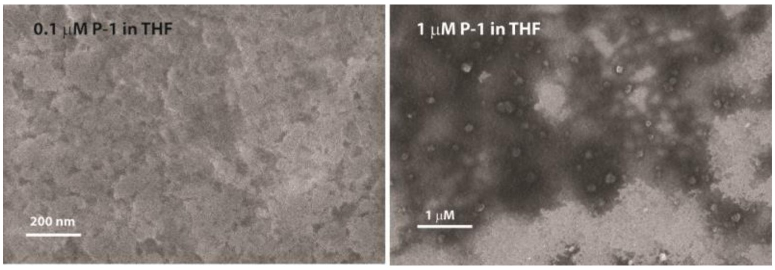

Figure S13. FESEM micrographs of **P-1** in THF at different concentrations.

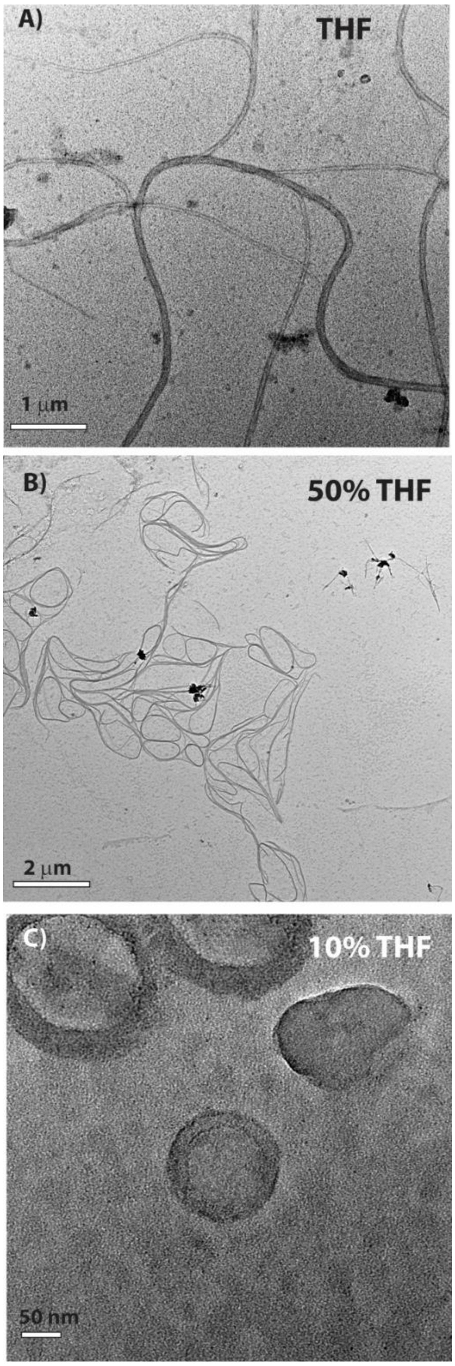

Figure S14. TEM micrographs of nanostructures formed by **P-1** in different THF-water compositions (10 μM) at room temperature. All measurements were carried out with 72 h matured samples.

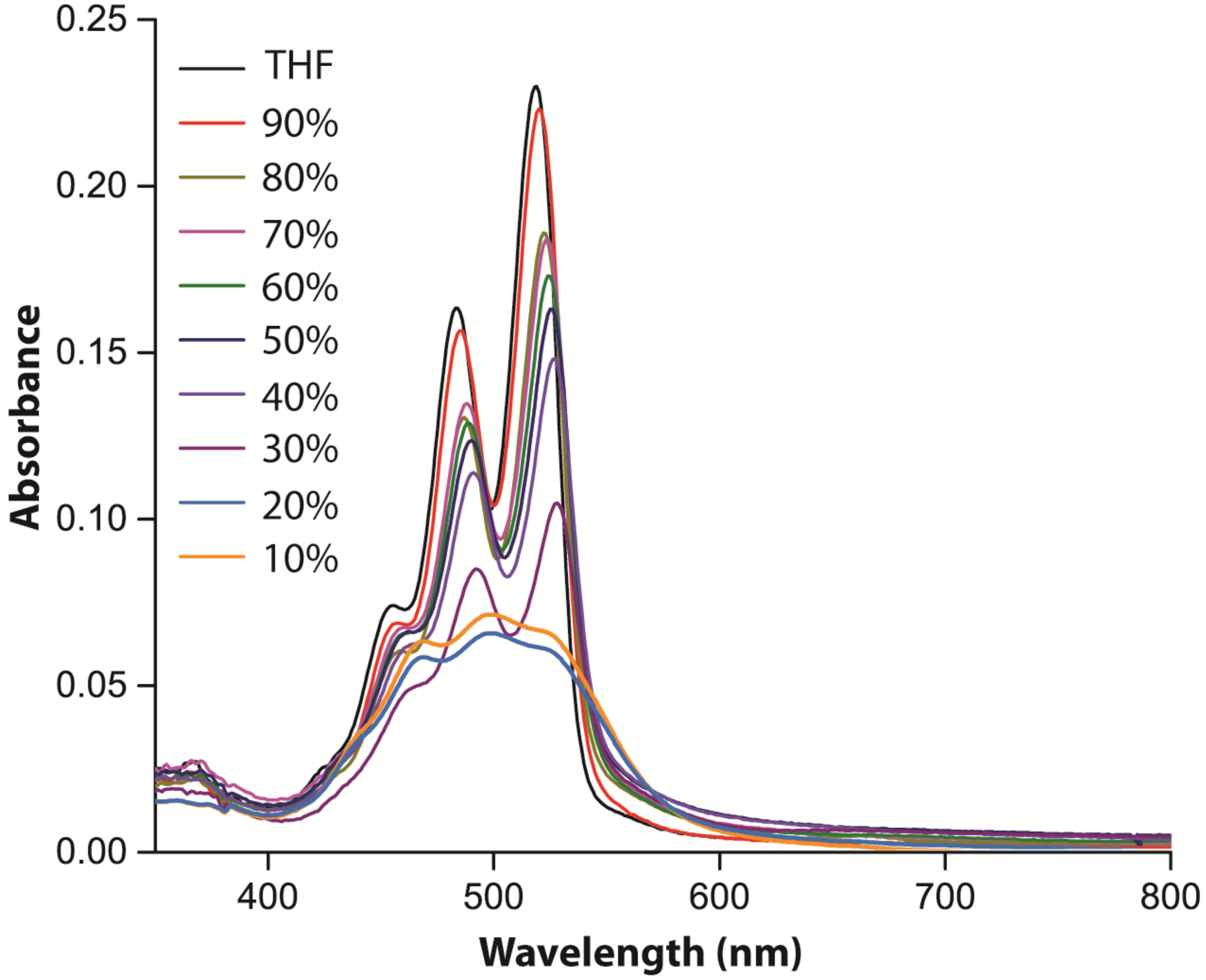

Figure S15. UV-Vis spectra of **P-1** (10 μM) in different THF-water compositions. All spectra were recorded using 72 h matured samples at room temperature.

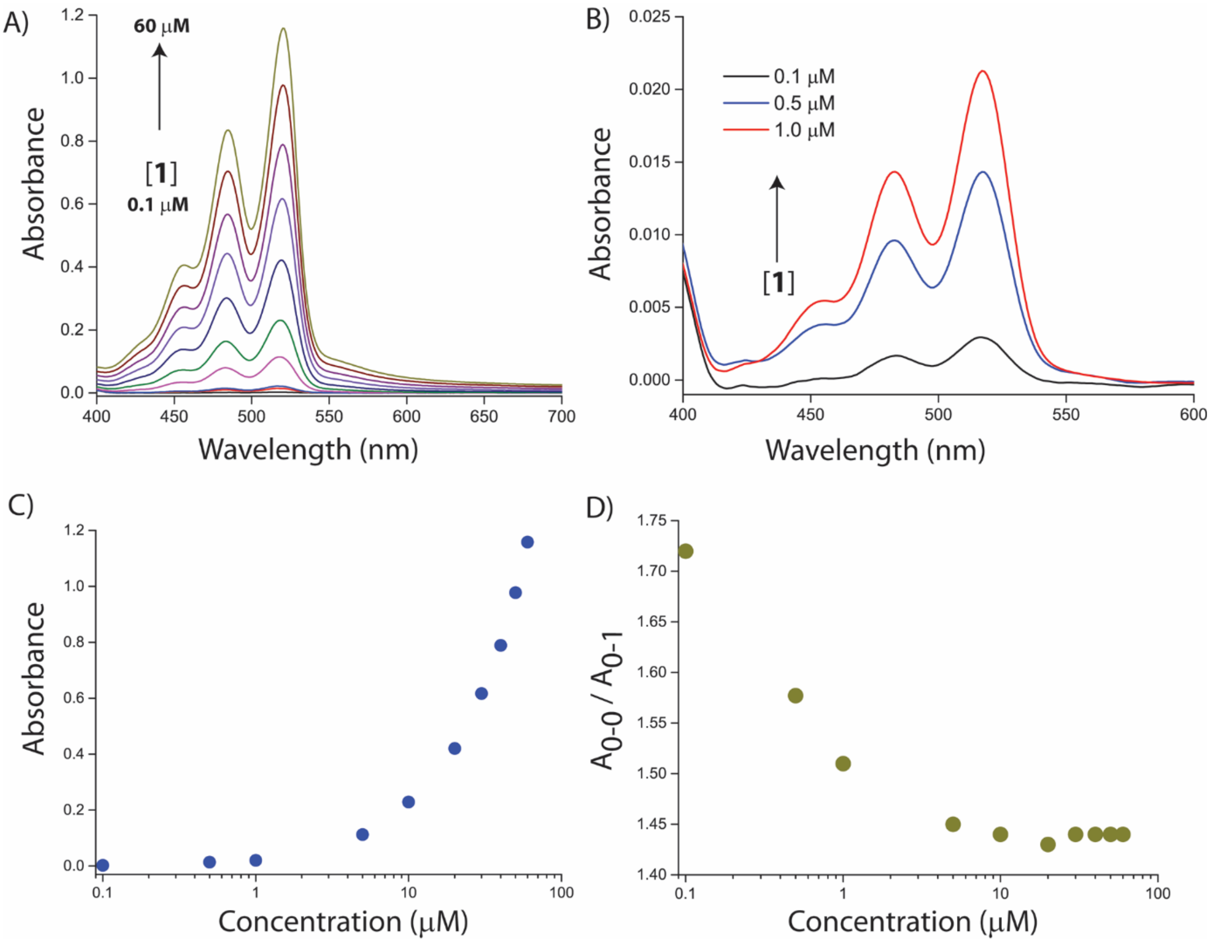

Figure S16. (A-B) Concentration dependent UV-Vis absorption spectra of **P-1** in THF; (C) dependence of the $A_{0\text{-}0}$ transition and (D) $A_{0\text{-}0}/A_{0\text{-}1}$ on concentration of **P-1** in THF. All measurements were carried out with 72 h matured samples at room temperature.

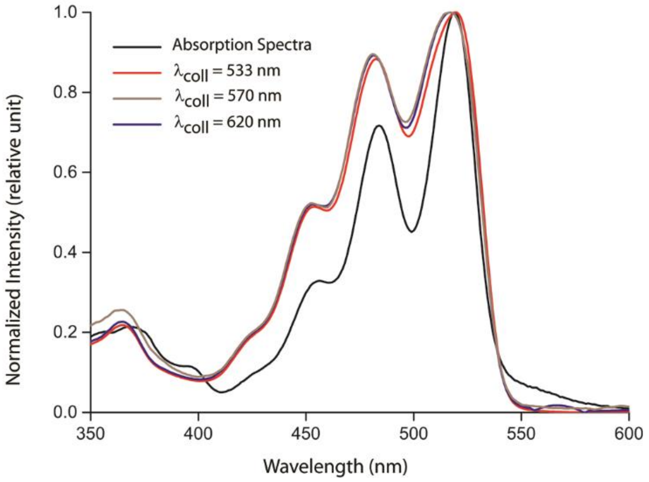

Figure S17. Excitation spectra of **P-1** in THF (10 μM) overlaid with the corresponding UV-Vis spectra.

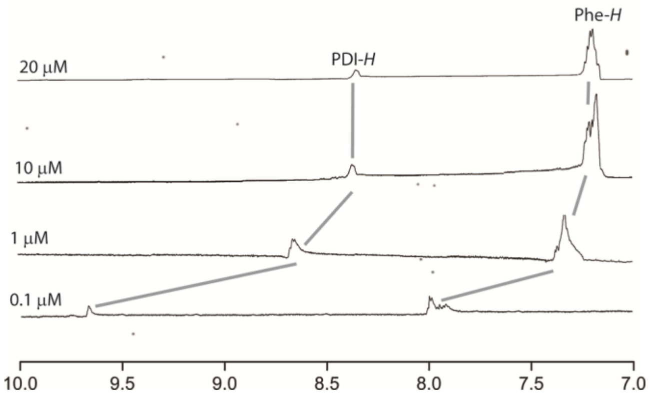

Figure S18. ^{1}H NMR spectra of **P-1** in THF-d_8 at different concentrations measured at room temperature.

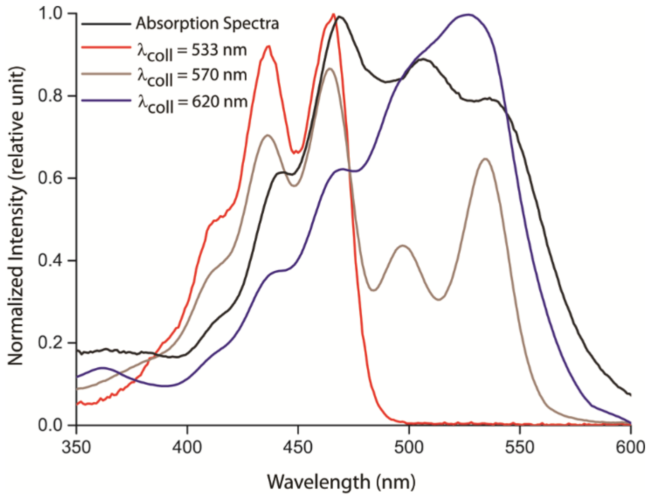

Figure S19. Excitation spectra of **P-1** in 10% THF (10 μM) overlaid with the corresponding UV-Vis spectra.

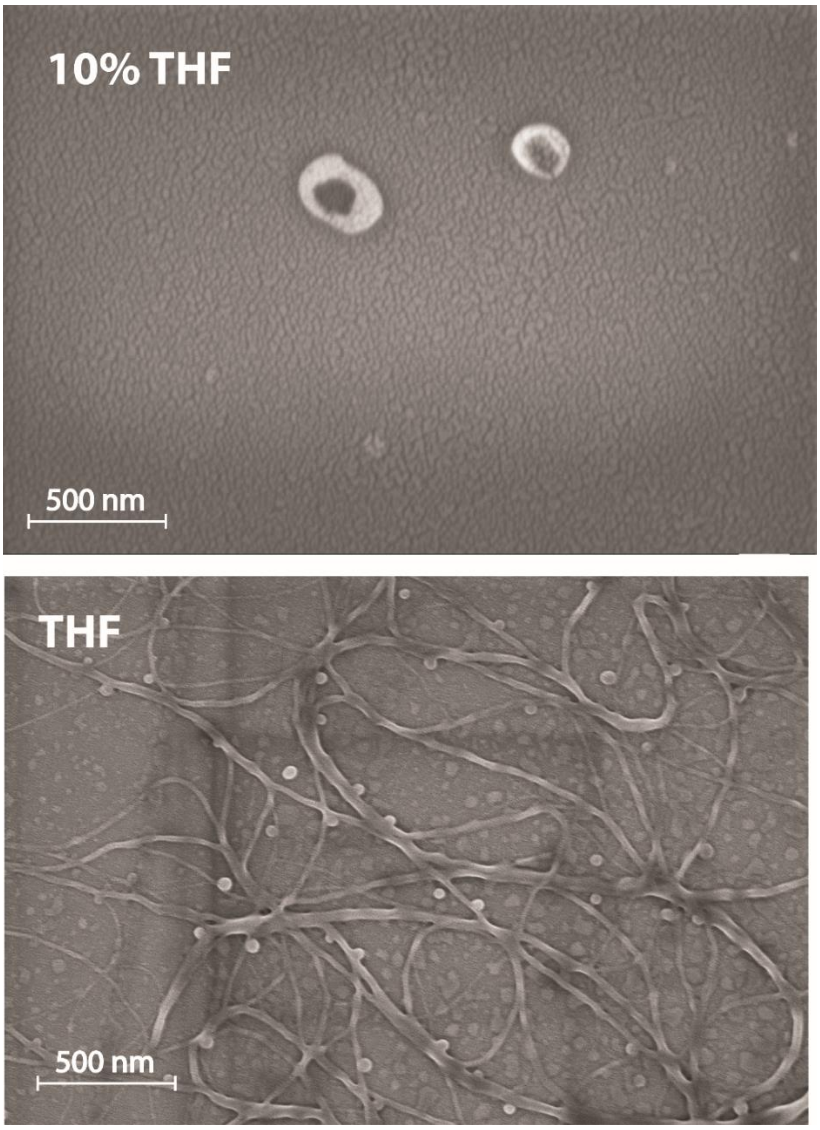

Figure S20. FESEM images of nanostructures formed by **D-P-1**in different THF-water compositions (10 μM) at room temperature. All measurements were carried out with 72 h matured samples.

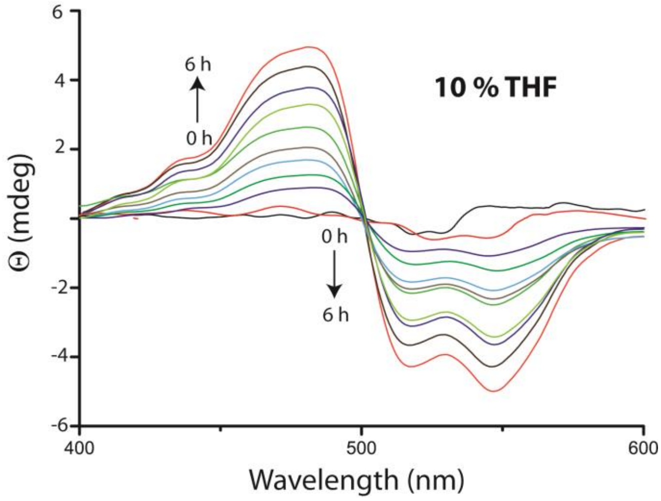

Figure S21. Time dependent (CD spectra of **P-1**(10 μM) in THF for 0-6 h.

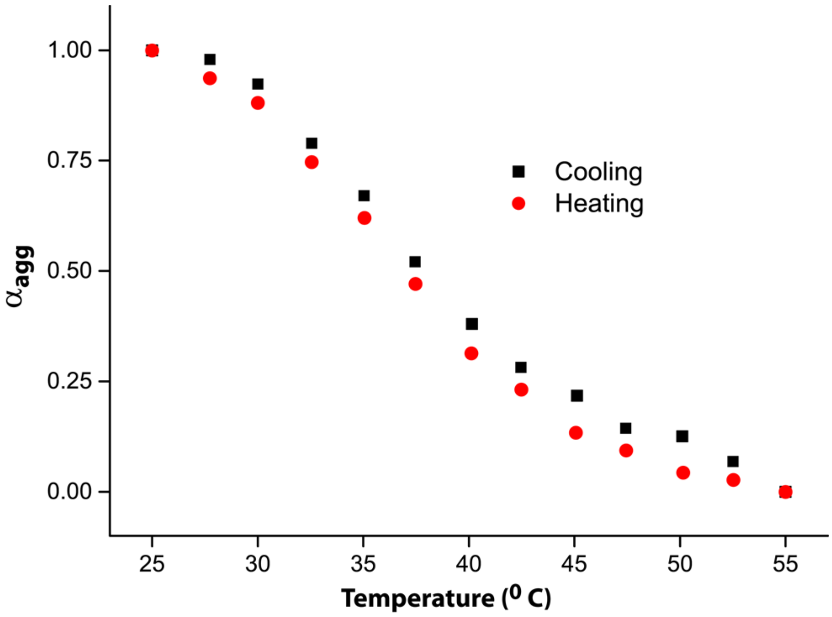

Figure S22. The mole-fraction of aggregate $\alpha_{agg}(T)$ as a function of temperature for **P-1** (10 μM) in 10% THF-water.

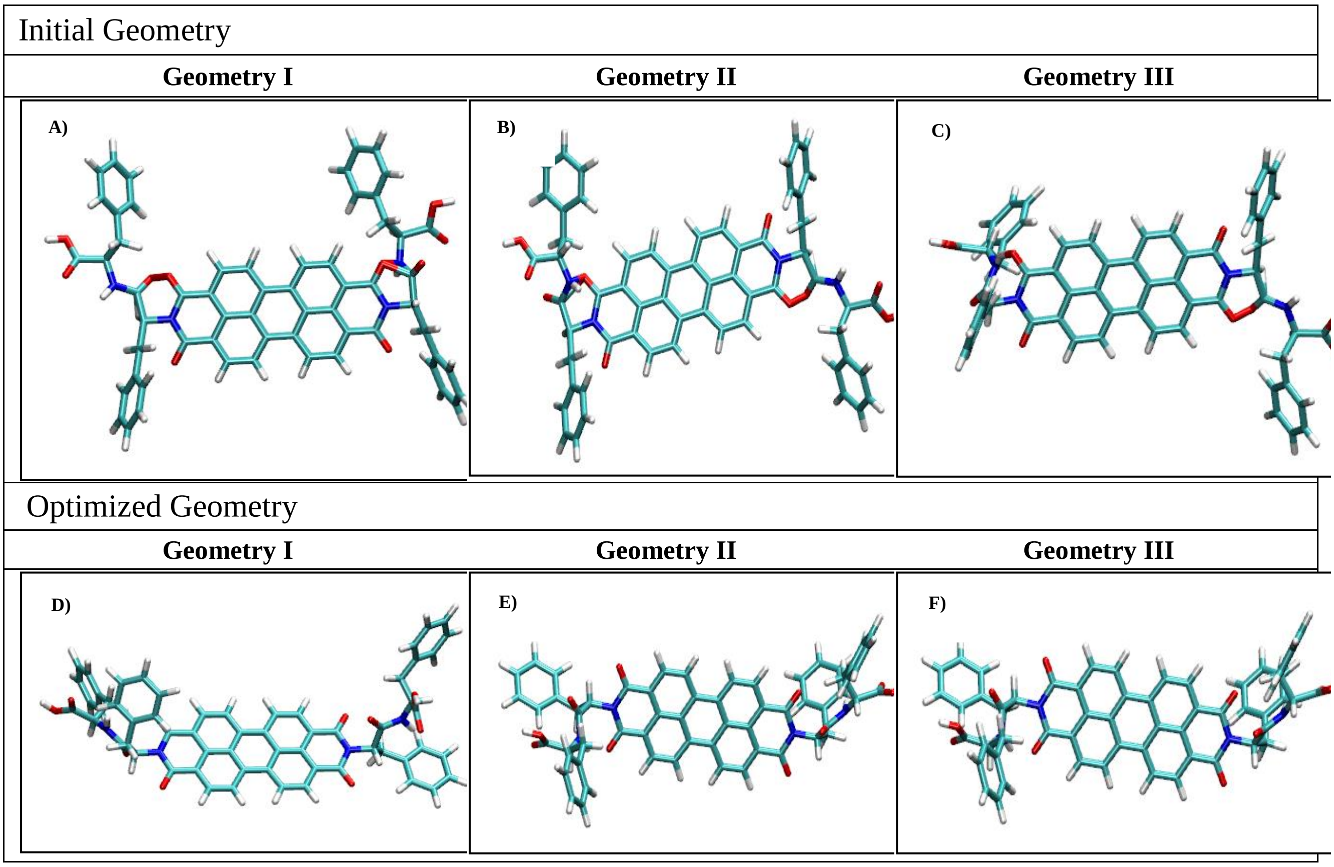

Figure S23. Three configurations of **P-1**molecule. (A)-(C) The initial geometries, (D)-(F) the optimized geometries respective to (A)-(C) .

Table S1. Final energies of the optimized structures of the three configurations. The energies are expressed in atomic units (a.u).

Geometry I	Geometry II	Geometry III
-3284.3365	-3284.3358	-3284.3357

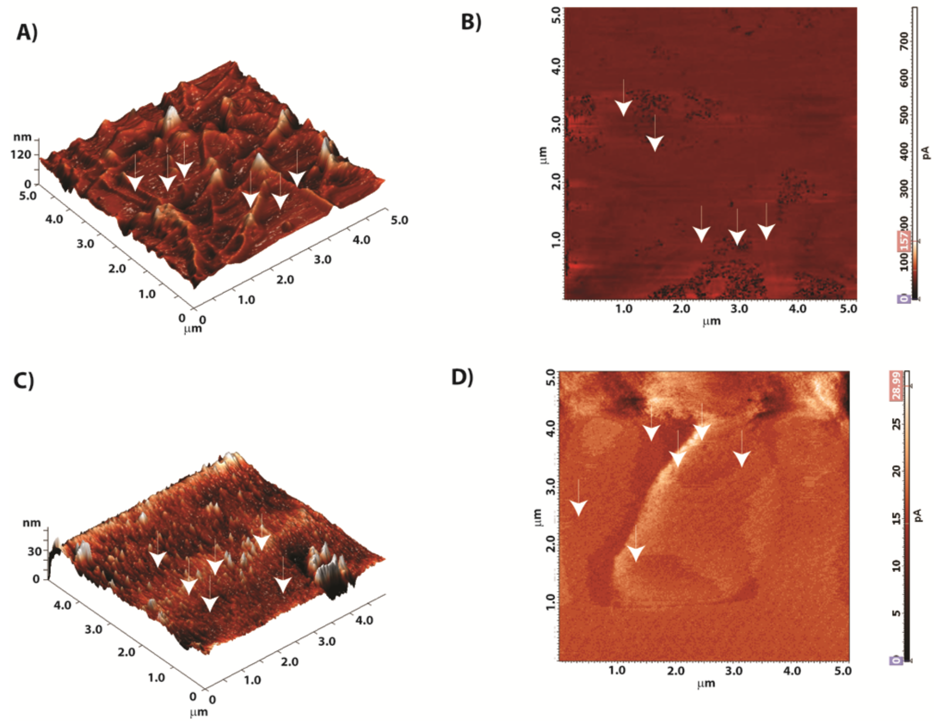

Figure S24. C-AFM analyses of thin film of **P-1** (10 μM) prepared from THF (A-B) and 10% THF (C-D). A) & C) 3D topography of the nanofibers and nano-rings respectively; B) & D) SRI pictures for the films; TheI-V characteristics for these positions are provided in the main manuscript.

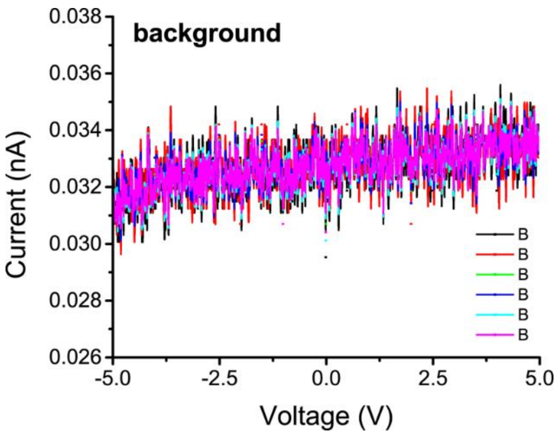

Figure S25. Background current-voltage characteristics from the doped Si surface. The background current-voltage characteristics were observed to be much lower and packed with noises as well as very much similar for both samples. These characteristics also do not have any specific trend.
